# Isomer-Dependent Pharmacokinetic Behavior and VKOR Interactions of Second-Generation Anticoagulant Rodenticides: An Integrated In Vivo–In Vitro–In Silico Investigation

**DOI:** 10.3390/ijms27093794

**Published:** 2026-04-24

**Authors:** Moyu Miyamae, Satoru Nagaoka, Teppei Hayama, Misaki Fukamatsu, Ryo Kamata, Kazuki Takeda

**Affiliations:** 1 Laboratory of Toxicology, School of Veterinary Medicine, Kitasato University, Towada 034-0021, Japan; 2 Daimaru Compound Chemical Co., Ltd., Nagano 381-1222, Japan; 3 School of Computing, Institute of Science Tokyo, Tokyo 152-8552, Japan

**Keywords:** second-generation anticoagulant rodenticides, cis–trans isomers, vitamin K epoxide reductase, protein–ligand interaction, molecular dynamics, molecular docking

## Abstract

Second-generation anticoagulant rodenticides (SGARs) were developed to overcome warfarin resistance in rodent populations; however, their prolonged hepatic retention has raised concerns regarding secondary poisoning of non-target wildlife. All major SGARs exist as cis–trans isomeric pairs, and differences in biological half-life between isomers have been reported, yet the molecular basis for such isomer-dependent pharmacokinetic behavior remains poorly understood. In this study, we conducted an integrated evaluation of cis and trans isomers of SGARs using in vivo, in vitro, and in silico approaches, with vitamin K epoxide reductase (VKOR) serving as the molecular target. The individual compounds exhibited distinct isomer-dependent profiles in hepatic retention, inhibitory potency (IC_50_), and VKOR interaction-related properties. Molecular dynamics simulations further revealed isomer-dependent differences in torsional flexibility around specific rotatable bonds and in ligand–VKOR interaction fractions. For flocoumafen and bromadiolone, the presence of an ether oxygen was associated with increased torsional and orientational flexibility and enhanced hydrogen-bonding potential, which may facilitate metabolic processing and contribute to the relatively faster elimination of cis isomers. Collectively, these results suggest that isomer-specific VKOR interaction patterns may contribute, in a compound-dependent manner, to isomer-dependent pharmacokinetic behavior, offering structural perspectives for the design of rodenticides with reduced ecological risk.

## 1. Introduction

Warfarin has long been used as a therapeutic agent for the prevention and treatment of thromboembolic disorders; however, it was originally developed as a rodenticide [1]. Because rodents serve as reservoirs or vectors for approximately 50 zoonotic pathogens, including Leptospira spp. and hantaviruses, population control using rodenticides remains an important public health measure worldwide [2,3]. Coumarin-type anticoagulant rodenticides exert their lethal effects by inhibiting vitamin K epoxide reductase (VKOR), thereby inducing fatal hemorrhage in rodents. Among blood coagulation factors, factors II, VII, IX, and X acquire their activity in a vitamin K-dependent manner [4]. In the liver, vitamin K (menaquinone) is reduced to its active hydroquinone form by vitamin K reductases, including ferroptosis suppressor protein 1 (FSP1) [5]. Vitamin K-dependent coagulation factors undergo γ-carboxylation of specific glutamate residues by γ-glutamyl carboxylase (GGCX), using vitamin K hydroquinone as a cofactor, which is essential for their biological activity [6]. During this process, vitamin K hydroquinone is oxidized to inactive vitamin K epoxide (VKO) and subsequently recycled back to vitamin K quinone by VKOR, constituting the vitamin K cycle [7,8]. By competitively inhibiting VKOR, warfarin-based rodenticides disrupt this cycle, leading to depletion of active coagulation factors and exerting anticoagulant effects [9].

Warfarin, a representative first-generation anticoagulant rodenticide, has been widely used since its introduction in the 1950s [10]. However, prolonged exposure has led to the emergence of resistant rodent populations worldwide, particularly in Europe, driven by mutations in the VKOR gene, making effective pest control increasingly difficult [11,12,13]. To overcome warfarin resistance, second-generation anticoagulant rodenticides (SGARs), such as difenacoum, were developed through chemical modification of the warfarin scaffold to enhance lipophilicity and biological persistence [14,15]. Despite their effectiveness, SGARs have raised serious environmental concerns due to their high bioaccumulative potential, with numerous reports of secondary poisoning in non-target wildlife, particularly raptors, in Europe and North America [16,17]. A Nature news report highlighted that SGARs, designed for rodent control, exhibit prolonged hepatic retention in predatory birds owing to their high lipophilicity and long biological half-lives [18]. In Canada, hepatic analyses of 196 raptors, including great horned owls (*Bubo virginianus*) and red-tailed hawks (*Buteo jamaicensis*), revealed frequent detection of SGAR residues. Importantly, modeling analyses suggested that toxic effects may occur even at hepatic concentrations below 0.1 mg/kg, a level previously considered to pose minimal risk, underscoring the underestimated threat of secondary poisoning [19]. Consequently, effective and environmentally safer strategies for controlling resistant rodent populations remain limited.

In recent years, increasing attention has been directed toward the cis–trans isomerism of SGARs as a potential strategy to retain efficacy while mitigating environmental impact. All five major SGARs exist as cis–trans isomeric pairs [20], and several studies have reported isomer-dependent differences in pharmacokinetics in rats as well as in accumulation profiles observed in wildlife under field conditions [21,22]. Damin-Pernik et al. (2017) demonstrated that, following a single oral administration of difenacoum (3.0 mg/kg) to male Sprague–Dawley rats, the trans isomer exhibited an approximately threefold shorter hepatic half-life than the cis isomer (trans: 24.18 h; cis: 78.33 h), indicating more rapid elimination of the trans isomer [23]. Notably, in vitro VKOR inhibition assays showed nearly identical inhibitory potency (Ki) between the cis and trans isomers (for difenacoum, trans: 21 ± 2; cis: 17 ± 1), leading to the conclusion that differences in pharmacokinetics, rather than intrinsic inhibitory activity, underlie the observed disparity in hepatic retention. Similar isomer-selective accumulation has been reported in non-rodent species. In wild red foxes (*Vulpes vulpes*) inhabiting France, the mean hepatic isomer composition of bromadiolone consisted of 99.7% trans and 0.3% cis, whereas difenacoum and difethialone were detected exclusively as the cis isomer [24]. Furthermore, hepatic analyses of wild boars (*Sus scrofa*) positive for difenacoum also revealed a predominance of the cis isomer [25]. Collectively, these multi-species observations strongly suggest that cis and trans isomers follow distinct metabolic and elimination pathways [26]. These findings indicate that subtle structural differences arising from isomerism can substantially influence the pharmacokinetic behavior of SGARs. However, the molecular determinants underlying enhanced elimination of specific isomers, as well as potential differences in VKOR binding between cis and trans isomers, remain unclear. In this study, we therefore aimed to investigate the molecular basis of isomer-dependent persistence by evaluating the binding properties of cis–trans isomers of five SGARs to their molecular target, VKOR, from structural and dynamic perspectives. We hypothesized that isomer-specific differences in molecular flexibility and VKOR interaction patterns may contribute to the observed differences in hepatic retention among SGARs.

VKOR is a four-transmembrane enzyme localized in the endoplasmic reticulum membrane [27], where it catalyzes substrate reduction via an electron relay mechanism involving two pairs of catalytic disulfide bonds (Cys43/Cys51 and Cys132/Cys135) [28,29]. During catalysis, VKOR adopts two major conformational states: an open conformation, characterized by disulfide bonds between Cys43–Cys51 and Cys132–Cys135, and a closed conformation, featuring a Cys51–Cys132 bridge [30]. The closed conformation is considered to represent the inhibitor-bound state of VKOR and thus serves as a relevant structural model for studying drug interactions [31,32]. The three-dimensional structure of VKOR was first elucidated in 2010 through X-ray crystallography of a VKOR homolog from *Synechococcus* [33], and the first experimental structure of human VKORC1 was determined in 2021 [31]. These advances have enabled detailed identification of the rodenticide-binding site on VKOR. Previous studies have shown that Tyr139 and Phe55 function as key interaction residues for warfarin binding [34], and mutations at these positions (e.g., Y139C/F/S) markedly reduce inhibitor affinity [12,35,36]. Resistance-associated mutations outside the canonical binding site have also been reported. For example, the Leu76Pro mutation frequently observed in roof rats (*Rattus rattus*) in Tokyo is located in an anchoring region distal to the binding pocket, yet in silico docking and molecular dynamics (MD) simulations have revealed that this mutation contributes to resistance by restricting the mobility of Phe55 [37,38]. These findings highlight the utility of in silico molecular simulations for elucidating VKOR–rodenticide interactions.

Accordingly, in the present study, we employed the closed conformation of VKOR to mimic the inhibitor-bound state and performed MD simulations to evaluate interaction stability and molecular flexibility between cis–trans isomers of SGARs. Furthermore, by integrating MD analyses with in vivo hepatic residue studies in rats and in vitro VKOR inhibition assays using HEK293T cells, we sought to elucidate the molecular mechanisms underlying isomer-dependent pharmacokinetic differences. The rodenticides examined in this study included two first-generation compounds, warfarin and diphacinone, and five widely used SGARs: difenacoum, brodifacoum, difethialone, flocoumafen, and bromadiolone.

## 2. Results

### 2.1. Hepatic Residue Profiles of Second-Generation Anticoagulant Rodenticides

**Figure 1:**
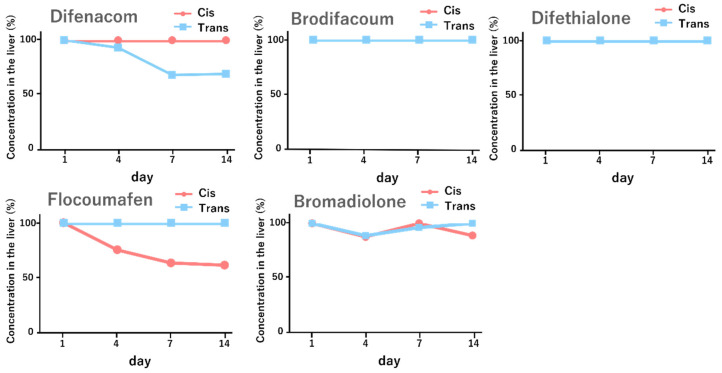
Time-dependent hepatic residue profiles of second-generation anticoagulant rodenticides determined by HPLC with UV detection. Each compound (difenacoum, brodifacoum, difethialone, flocoumafen, or bromadiolone) was administered separately as a cis/trans isomer mixture at ratios of 1:1, 6:4, 1:1, 2:8, and 8:2, respectively, at a dose of 0.1 mg/kg. Livers were collected on days 1, 4, 7, and 14 after administration and analyzed by HPLC with UV detection (*n* = 2). Representative chromatograms supporting cis/trans peak assignment are shown in Supplementary Figure S7, and the corresponding retention-time, chromatographic resolution, and calibration data are summarized in Supplementary Table S4. Hepatic concentrations of individual isomers are expressed as relative values (%) normalized to the concentration of each isomer on day 1. Red circles denote cis isomers, and blue squares denote trans isomers.

After each second-generation anticoagulant rodenticide—difenacoum, brodifacoum, difethialone, flocoumafen, or bromadiolone—was administered as a cis/trans isomeric mixture, livers were collected at multiple time points. Hepatic concentrations of individual isomers were quantified by HPLC with UV detection and expressed as relative concentrations (%) within each compound; representative chromatograms supporting cis/trans peak assignment are provided in Supplementary Figure S7, and the corresponding retention-time, chromatographic resolution, and calibration data are summarized in Supplementary Table S4. The resulting time-dependent hepatic residue profiles are shown in Figure 1. Accordingly, the hepatic residue dataset was used primarily to compare within-compound temporal changes in cis/trans residue patterns rather than to make standardized quantitative comparisons across different SGARs. Among the tested compounds, difenacoum showed a decrease in the relative hepatic concentration of the trans isomer over time, whereas the cis isomer remained comparatively stable. In contrast, flocoumafen exhibited an earlier decline in the relative hepatic concentration of the cis isomer.

### 2.2. Inhibition of Factor IX Activity by First- and Second-Generation Rodenticides

**Figure 2:**
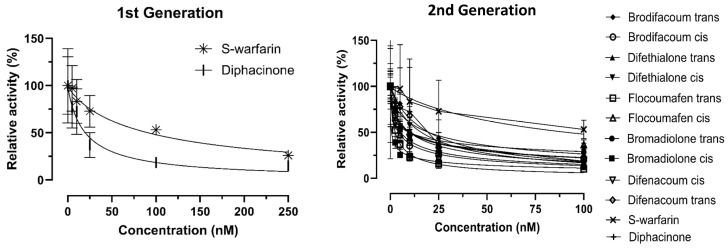
Inhibition of Factor IX activity by first- and second-generation rodenticides in HEK293T cells expressing *Rattus norvegicus* VKOR. Cells were treated with first-generation rodenticides (S-warfarin and diphacinone) at final concentrations of 0, 5, 10, 25, 100, and 250 nM, and with cis and trans isomers of second-generation rodenticides (brodifacoum, difethialone, flocoumafen, bromadiolone, and difenacoum) at final concentrations of 0, 2.5, 5, 10, 25, and 100 nM (*n* = 4). The *x*-axis represents inhibitor concentration, and the *y*-axis represents FIX activity (%). Error bars indicate the standard error of the mean (SEM). Symbols indicate the following compounds and isomers: * warfarin; + diphacinone; ◆ brodifacoum (trans); ○ brodifacoum (cis); ▲ difethialone (trans); ▼ difethialone (cis); □ flocoumafen (trans); △ flocoumafen (cis); ● bromadiolone (trans); ■ bromadiolone (cis); ▽ difenacoum (cis); ◇ difenacoum (trans).

The inhibitory effects of cis and trans isomers of each rodenticide on Factor IX (FIX) activity were evaluated (Figure 2). For all tested compounds, FIX activity decreased in a concentration-dependent manner. The IC_50_ values calculated from the respective concentration–response curves are summarized in Table 1. Among the second-generation rodenticides, brodifacoum exhibited a lower IC_50_ value for the cis isomer than for the trans isomer, corresponding to approximately threefold stronger inhibitory potency based on the IC_50_ values. In contrast, difethialone showed no substantial difference in inhibitory potency between the cis and trans isomers. For flocoumafen, the cis isomer (2.2 ± 0.2 nM) exhibited a significantly lower IC_50_ value than the trans isomer (7.5 ± 0.1 nM) (*p* < 0.05). In bromadiolone and difenacoum, differences in IC_50_ values between cis and trans isomers were observed; however, these differences did not reach statistical significance. The reference first-generation rodenticides, warfarin and diphacinone, exhibited IC_50_ values of 21.9 ± 0.3 nM and 15.5 ± 0.2 nM, respectively. All second-generation rodenticides showed stronger inhibitory activity toward FIX than these first-generation compounds. Preliminary evaluation of FIX activity inhibition by cis and trans isomers of difenacoum is shown in Figure S1.

### 2.3. Comparative Docking Analysis of Cis and Trans Isomers of Second-Generation Rodenticides in VKOR

**Figure 3:**
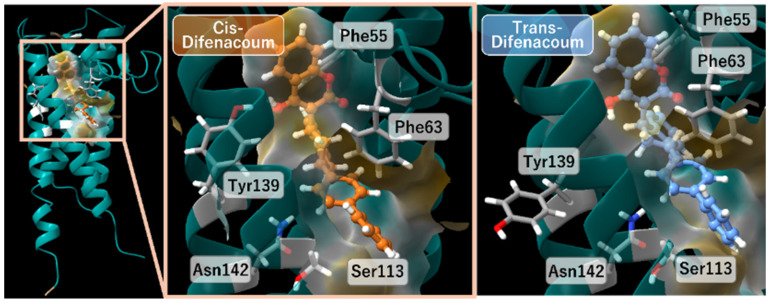
Binding poses of cis and trans difenacoum in the closed conformation of *Rattus norvegicus* VKOR. The cyan ribbon represents the three-dimensional structure of VKOR from *Rattus norvegicus* in the closed conformation. The left panel shows the overall VKOR structure, and the center panel presents an enlarged view of the binding site with cis-difenacoum shown in orange. The right panel shows the binding pose of trans-difenacoum in blue, rendered at the same orientation and scale. Regions within 5 Å of the ligand are displayed as a semi-transparent molecular surface. Key interacting residues (Phe55, Phe63, Tyr139, Ser113, and Asn142) are shown in gray. Atom colors are as follows: white, hydrogen; red, oxygen; gray, carbon; and blue, nitrogen.

**Figure 4:**
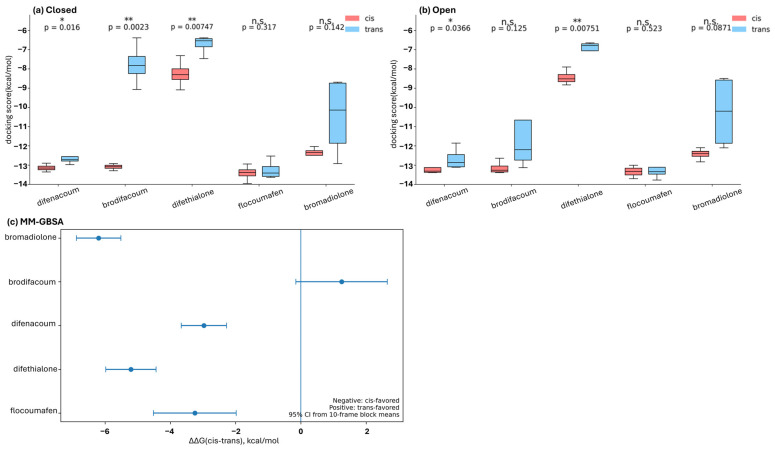
Comparison of cis/trans docking score tendencies and MM-GBSA results for second-generation anticoagulant rodenticides. (**a**) Comparison of docking scores obtained for the closed-state VKOR models from four species (Rattus norvegicus, Rattus rattus, Mus musculus, and Homo sapiens). (**b**) Comparison of docking scores obtained for the open-state VKOR models from the same four species. In panels (**a**,**b**), docking scores were used as comparative indices of pose favorability under identical docking conditions, with lower values indicating more favorable predicted docking poses within the predefined binding pocket. Red and blue boxes represent cis and trans isomers, respectively. Data are shown as the mean ± SEM (*n* = 4 per conformational state). (**c**) MM-GBSA results obtained from the rat-centered MD workflow using induced-fit-refined complexes based on the closed-state Rattus norvegicus VKOR model. Lower MM-GBSA values indicate more favorable estimated binding energies under the present calculation conditions. Statistical significance between cis and trans isomers is indicated as follows: * *p* < 0.05; ** *p* < 0.01; n.s., not significant. RMSD comparisons relative to the OPLS4-minimized input ligand conformations are provided separately in Supplementary Figure S2.

Molecular docking simulations were performed for five second-generation anticoagulant rodenticides using three-dimensional structures of vitamin K epoxide reductase (VKOR) from four species (*Rattus norvegicus*, *Rattus rattus*, *Mus musculus*, and *Homo sapiens*) (Figure 3). For each VKOR, docking calculations were conducted separately for the open and closed conformations, and cis/trans docking score tendencies were compared within each conformational state (Figure 4a,b). As a result, difenacoum and difethialone exhibited significantly lower docking scores for the cis isomers than for the trans isomers (*p* < 0.001), indicating more favorable predicted docking poses for the cis isomers under the present docking conditions. In contrast, no significant difference in docking scores between cis and trans isomers was observed for flocoumafen (*p* = 0.191). These results indicate that cis/trans docking score tendencies differed among compounds.

To further assess whether the cis/trans docking tendencies observed in the AF2-based VKOR models were broadly compatible with experimentally resolved VKOR structures, supplementary docking analyses were performed using available human VKOR crystal structures (PDB IDs: 6WV3 and 6WVH), and the resulting cis/trans rankings were compared with those obtained from the AF2-based models (Supplementary Table S1). For difenacoum, brodifacoum, and bromadiolone, the cis isomer consistently showed more favorable docking scores than the trans isomer across all examined structures, whereas difethialone showed a predominantly cis-favored tendency with one exception, and flocoumafen showed structure-dependent behavior. Overall, these supplementary comparisons indicate that the AF2-based models reproduced the relative cis/trans docking tendency for several compounds, while also revealing compound-dependent structural variability. In addition, structural superposition of the docking-ready closed-state AF2-derived human VKOR model and the experimentally resolved human VKOR structure yielded an RMSD of 0.645 Å for 149 atom pairs after automatic exclusion of poorly fitting pairs (pruning) and 2.475 Å for all 158 matched atom pairs without pruning, indicating close agreement in the aligned core region while larger deviations were observed when more structurally variable peripheral regions were included. Furthermore, to assess the structural validity of the most favorable binding poses obtained from docking, RMSD (root mean square deviation) analyses were performed using energy-minimized docking poses as the initial conformations (Figure S2). As in the docking score analysis, both open and closed VKOR structures were included, and RMSD values were compared between cis and trans isomers for each compound. Difenacoum, brodifacoum, and difethialone showed significantly lower RMSD values for the cis isomers than for the trans isomers (*p* < 0.05), indicating lower structural deviation from the corresponding input poses for the cis isomers. In contrast, flocoumafen exhibited significantly lower RMSD values for the trans isomer (*p* < 0.001), indicating lower structural deviation from the corresponding input pose for the trans isomer. Specifically, RMSD values for the cis isomer ranged from approximately 8.0 to 10.3 Å, whereas those for the trans isomer ranged from approximately 5.9 to 9.4 Å, corresponding to an average difference of approximately 2 Å. For bromadiolone, no statistically significant difference was observed; however, the trans isomer tended to show lower RMSD values than the cis isomer.

### 2.4. MD-Based Analysis of Cis and Trans Isomers of Second-Generation Rodenticides in Rattus norvegicus VKOR

Molecular dynamics (MD) simulations were performed for 100 ns for complexes of cis and trans isomers of second-generation anticoagulant rodenticides bound to vitamin K epoxide reductase (VKOR) from *Rattus norvegicus*. For the rat-centered MD analysis, the initial VKOR–ligand complexes were prepared by induced-fit docking using the closed-state R. norvegicus VKOR model, allowing local receptor–ligand accommodation prior to simulation. To further examine whether the cis/trans tendencies suggested by the initial docking analysis were retained after refinement in the rat-centered workflow, MM-GBSA analyses were performed using the MD trajectories (Figure 4c). The MM-GBSA results showed cis-favored energetic tendencies for bromadiolone, difenacoum, difethialone, and flocoumafen, whereas brodifacoum showed a trans-favored tendency. Thus, the MM-GBSA analysis partly supported the cis/trans ranking suggested by docking, while also revealing compound-dependent concordance and discordance among the analyzed compounds.

**Figure 5:**
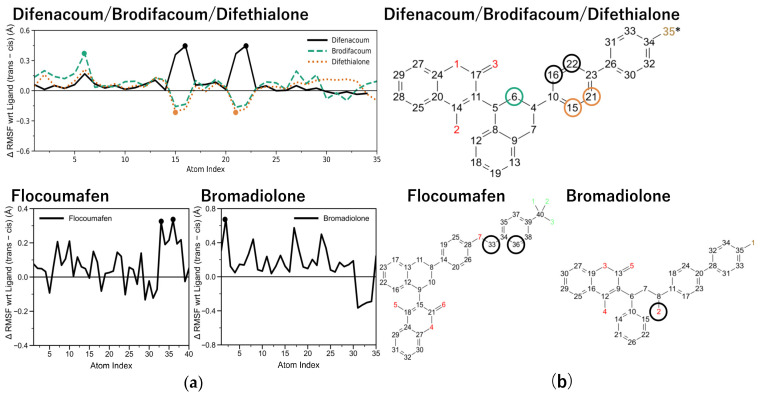
Ligand flexibility differences between cis and trans isomers in complexes of second-generation rodenticides with *Rattus norvegicus* VKOR analyzed by L-RMSF. (**a**) ΔRMSF profiles showing differences in ligand atomic fluctuations between cis and trans isomers. The *y*-axis represents ΔRMSF values (L-RMSF_trans− – L-RMSF_cis), and the *x*-axis represents ligand atom indices. Results for difenacoum (black line), brodifacoum (green line), and difethialone (orange line) are shown in the upper panels, while results for flocoumafen (lower left) and bromadiolone (lower right) are shown in the lower panels. (**b**) Correspondence between ligand atom indices used in the MD simulations and positional numbering on the two-dimensional chemical structures of each ligand. Key atom indices showing pronounced cis/trans differences in flexibility are highlighted with circles in panel (**a**) and on the corresponding chemical structures in panel (**b**). Atom indices exhibiting cis/trans-dependent differences in flexibility are annotated on the chemical structures. Atom indices correspond to the fixed atom numbering used in the L-RMSF output generated by Schrödinger/Desmond. Carbon (C) atoms are shown in black, oxygen (O) atoms in red, bromine (Br) substituents in ocher, sulfur (S) atoms in yellow, and fluorine (F) atoms in green. The upper panels show the chemical structures of difenacoum, brodifacoum, and difethialone. Difenacoum consists of a core scaffold corresponding to positions 1–34, excluding the side chain at position 35. Brodifacoum contains a bromine (Br) substituent at position 35. Difethialone also contains a substituent at position 35 but is characterized by substitution of the oxygen atom at position 1 with a sulfur atom. Asterisks indicate the positions used to focus on the presence or absence of a side chain, highlighting the comparison between compounds lacking a side chain, such as difenacoum, and those bearing a side chain, such as brodifacoum and difethialone. The lower panels show the chemical structures of flocoumafen (**left**) and bromadiolone (**right**).

Ligand atomic fluctuations were evaluated based on the MD trajectories by calculating ligand root mean square fluctuation (L-RMSF) values for each ligand atom. Differences in ligand flexibility between cis and trans isomers (ΔRMSF), as defined in Equation (1), are shown in Figure 5a. Positive ΔRMSF values indicate greater atomic fluctuations in the trans isomer, whereas negative values indicate greater fluctuations in the cis isomer. Detailed L-RMSF profiles for individual cis and trans isomers are provided in Supplementary Figure S3. As a result, differences in flexibility between cis and trans isomers were observed at specific ligand atoms, as identified by qualitative comparison of the ΔRMSF profiles. Specifically, such differences were observed at atoms 16 and 22 for difenacoum, atom 6 for brodifacoum, atoms 15 and 21 for difethialone, atoms 33 and 36 for flocoumafen, and atom 2 for bromadiolone. These ΔRMSF peaks were interpreted as atom-wise indicators of cis/trans-dependent ligand flexibility and were not assumed to imply a strict one-to-one correspondence between a single fluctuating atom and a single persistent residue contact in the contact schematics.

### 2.5. Identification of VKOR Residues Associated with Ligand Fluctuations in Second-Generation Rodenticides

**Figure 6:**
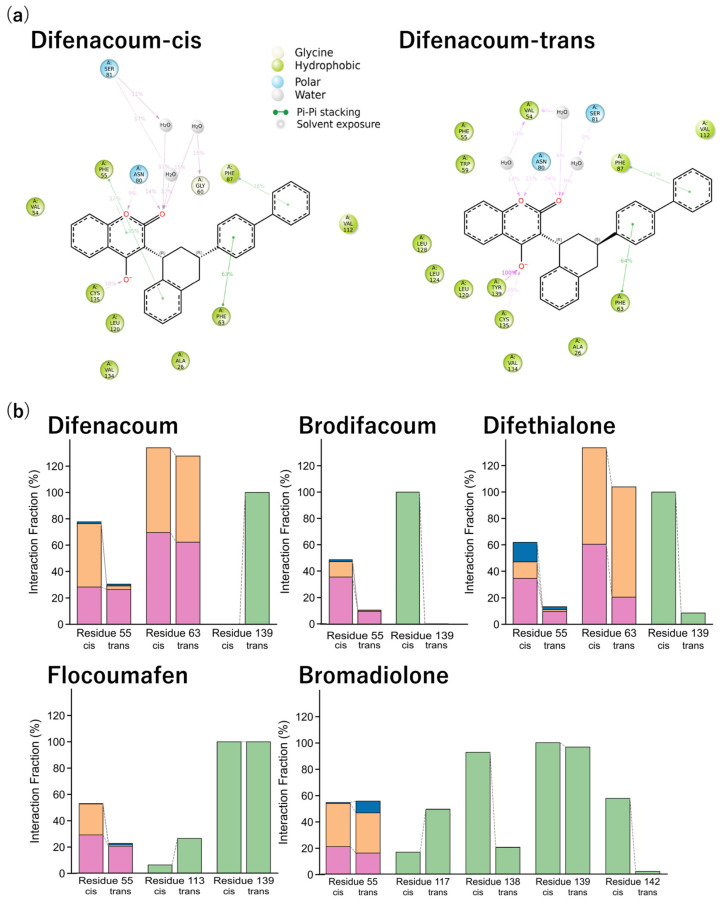
Protein–ligand interaction analysis between rat VKOR and cis/trans isomers of difenacoum. (**a**) Atom-wise ligand–protein contact schematics derived from the SID Ligand–Protein Contacts output for the cis (**left**) and trans (**right**) isomers of difenacoum bound to rat VKOR. Hydrophobic residues are shown in yellow–green and polar residues in light blue. Red indicates oxygen atoms (O). White spheres represent water-mediated interactions. Pink lines indicate non-covalent polar interactions, primarily hydrogen bonds, while green lines indicate π–π interactions. The numerical values associated with each interaction represent the interaction fraction (%) during a 100 ns MD simulation. For example, a value of 91% indicates that the interaction was observed during 91% of the total simulation time. Only interactions occurring for more than 5% of the simulation time are displayed in this panel. Difenacoum is shown as a representative example; corresponding results for other second-generation anticoagulant rodenticides are provided in Supplementary Figure S4. (**b**) Interaction fraction analysis between rat VKOR residues and the cis/trans isomers of difenacoum. Green bars indicate hydrogen bonds, pink bars indicate hydrophobic interactions, orange bars indicate π–π stacking, and blue bars indicate water bridges. The vertical axis represents the interaction fraction (%), indicating the percentage of the simulation time during which each interaction was maintained. The horizontal axis represents VKOR amino acid residues involved in the interactions. Dashed lines are included to visually emphasize differences between the cis and trans isomers. Because panels (**a**,**b**) represent different levels of summarization, they should not be interpreted as a strict one-to-one atom/residue mapping. A complete interaction fraction dataset for all residues is provided in Supplementary Table S2.

Ligand atoms exhibiting differential fluctuations between cis and trans isomers were identified by ΔRMSF analysis based on a comparison of all ligand atoms (Figure 5a,b). Using these atoms, PL_Contact analysis was conducted to examine the residue environments sampled by the corresponding ligand regions in rat VKOR (Figure 6a). Representative interaction diagrams for the cis and trans isomers of difenacoum are shown in Figure 6a, while the results for the other second-generation anticoagulant rodenticides are presented in Supplementary Figure S4. Because the SID outputs used here include both residue-wise interaction summaries and filtered atom-wise ligand–protein contact schematics, these analyses were interpreted as complementary representations rather than as strict one-to-one atom-to-residue mappings for every ΔRMSF peak. Based on this analysis, difenacoum and difethialone showed prominent Phe63/Phe87-centered hydrophobic contact environments, whereas flocoumafen and bromadiolone showed broader mixed interaction environments that included both hydrophobic contacts and polar or water-mediated contacts involving residues such as Ser113, Ser117, Thr138, Asn80, and Ser81 depending on the isomer. In addition to these fluctuation-associated residues, interaction fractions were calculated for all VKOR residues, including Phe55 and Tyr139, which have been previously reported to be involved in warfarin binding (Figure 6b). Detailed interaction fraction data for all residues are provided in Supplementary Table S2. For difenacoum, hydrophobic and π–π interactions with Phe63 were maintained with interaction fraction of 69% and 64%, respectively, in the cis isomer, whereas these values decreased to 62% and 62% in the trans isomer. In multiple second-generation anticoagulant rodenticides, lower contact persistence, as reflected by reduced interaction fractions, was observed in the trans isomers at VKOR residues interacting with ligand atoms that exhibited ΔRMSF differences. In contrast, flocoumafen was an exception, in which reduced interaction fractions were observed in the cis isomer.

### 2.6. Evaluation of Internal Conformational Flexibility of Second-Generation Anticoagulant Rodenticides by L-Torsion Analysis

**Figure 7:**
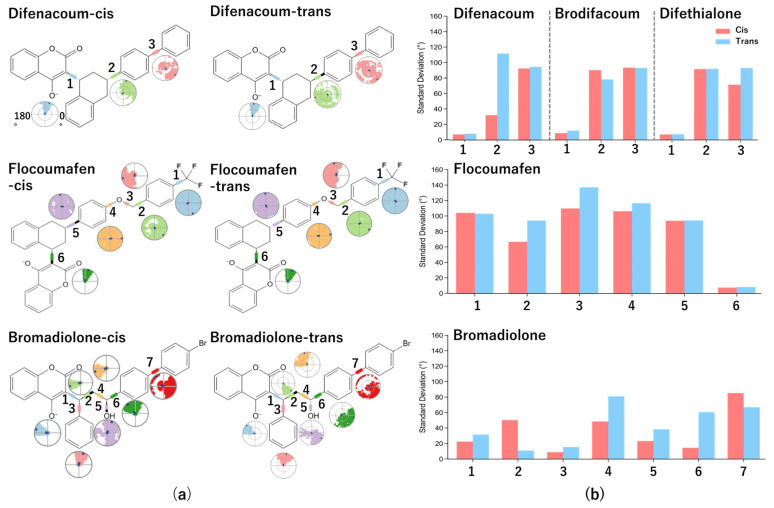
Comparison of internal conformational flexibility between cis and trans Isomers of Second-Generation Anticoagulant Rodenticides by L-Torsion Analysis. (**a**) Chemical structures of second-generation anticoagulant rodenticides highlighting the positions of rotatable bonds (Torsion1–7). Each torsion axis is color-coded, and the corresponding dial plots depict the temporal evolution of dihedral angles over the MD simulation (0–100 ns). The center of each plot represents the initial simulation time point, with increasing radial distance indicating temporal progression. These torsion axes were selected as target regions for evaluating internal ligand flexibility. (**b**) Standard deviations (°) of dihedral angles for each torsion axis (Torsion1–7) of rodenticide ligands in complex with *Rattus norvegicus* VKOR. The vertical axis represents the standard deviation of torsion angles, and the horizontal axis indicates the torsion index. Red bars denote cis isomers, and blue bars denote trans isomers. Detailed torsion angle distributions are provided in Supplementary Figure S5. Torsion indices exhibiting pronounced cis/trans differences are highlighted in bold and underlined in both panels (**a**,**b**).

For each second-generation anticoagulant rodenticide, the standard deviations of dihedral angles around internal rotatable bonds (Torsion1–7) were calculated from molecular dynamics (MD) simulations of ligand–VKOR complexes (100 ns, 2500 frames) to characterize ligand internal flexibility (Figure 7). Detailed distributions of individual torsion angles are provided in Supplementary Figure S4. Distinct rotational behaviors were observed across torsion axes within each compound. In difenacoum, pronounced fluctuations were detected at Torsion3, corresponding to the side-chain region, in both cis and trans isomers. In contrast, torsion axes associated with the coumarin core exhibited minimal differences between cis and trans isomers across all compounds analyzed. Flocoumafen and bromadiolone exhibited characteristic patterns distinct from the other rodenticides. In these compounds, a greater number of torsion axes showed relatively large standard deviations compared with the remaining compounds, indicating broader distributions of internal rotational motion. In flocoumafen, the trans isomer displayed larger standard deviations at Torsion2–4, corresponding to the aromatic ring region, whereas reduced fluctuations at these torsion axes were observed in the cis isomer. In bromadiolone, standard deviations were relatively low across many torsion axes compared with the other compounds. Nevertheless, at Torsion1, 3, 4, 5, and 6, the trans isomer consistently exhibited larger fluctuations than the cis isomer. Overall, the distribution and extent of internal rotational flexibility varied among second-generation anticoagulant rodenticides and differed between cis and trans isomers within individual compounds.

## 3. Discussion

Previous wildlife monitoring studies have repeatedly reported isomer-selective hepatic residue patterns for second-generation anticoagulant rodenticides (SGARs), indicating that cis and trans isomers do not necessarily show the same hepatic retention patterns under field conditions. In a field survey of wild *Rattus norvegicus* captured in Paris, France, commercial baits containing cis/trans mixtures resulted in highly skewed hepatic isomer profiles. Specifically, bromadiolone residues consisted exclusively of the trans isomer in five of seven animals (trans-bromadiolone, 100%), whereas difenacoum residues were composed solely of the cis isomer in nine of twelve animals (cis-difenacoum, 100%), with the trans isomer being nearly below the limit of detection [39]. Similar isomer-selective patterns were observed in a hepatic residue analysis of the Réunion harrier (*Circus maillardi*), in which cis isomers predominated for difenacoum, brodifacoum, and difethialone. Notably, difenacoum residues consisted entirely of the cis isomer (20.6 ± 19.5 ng/g ww; cis-difenacoum 100 ± 0%, trans-difenacoum 0 ± 0%), whereas bromadiolone residues were almost exclusively trans-enriched [21]. In addition, blood monitoring studies in European vultures and kites have demonstrated compound-specific isomer preferences, with trans isomers predominating for flocoumafen (cis 13%, trans 87%) and bromadiolone (cis 1%, trans 99%), while cis isomers were dominant for difenacoum (cis 93%, trans 7%) and brodifacoum (cis 86%, trans 14%) [40]. Collectively, these observations suggest that isomer-dependent metabolism and/or elimination processes may operate in vivo, leading to selective hepatic retention of one isomer over the other under environmental exposure scenarios. When compared with the present in vivo experiments, the reduced hepatic retention observed for trans-difenacoum and cis-flocoumafen was consistent with residue patterns reported in previous studies. These results indicate that the present findings are in agreement with isomer-selective hepatic residue patterns observed under wildlife exposure conditions, and suggest that the experimental system employed here is useful for examining factors contributing to such isomer-selective hepatic retention.

To explore the molecular factors potentially underlying isomer-selective hepatic retention, the present study employed in silico analyses to examine the binding modes and relative structural tendencies of VKOR complexes formed with each SGAR isomer. Prior to detailed analysis, the structural stability of the MD simulations was evaluated based on both protein backbone RMSD and ligand RMSD profiles. Across all complexes, protein backbone RMSD increased during the initial equilibration phase and subsequently reached a plateau, indicating stable conformational behavior. Ligand RMSD exhibited compound- and isomer-dependent fluctuations, including transient increases consistent with ligand pose relaxation or reorientation within the binding pocket, followed by re-stabilization. Importantly, no trajectories showed continuous or unbounded RMSD increase, confirming that all simulated systems maintained stable structural behavior throughout the simulations (Figure S6). With this structural stability established, the computational and experimental cis/trans trends were compared across compounds, both concordant and discordant patterns were observed (Table S5). Specifically, difenacoum showed the broadest overall concordance between hepatic retention and the in silico metrics, whereas brodifacoum, difethialone, flocoumafen, and bromadiolone showed partial, limited, or mixed agreement depending on the metric considered. These results indicate that no single computational metric consistently reproduced the experimental cis/trans behavior across all SGARs. For difenacoum, reduced hepatic retention was observed for the trans isomer. This tendency was broadly consistent with the computational results, in which the cis isomer showed more favorable docking score tendencies, lower structural deviation from the input ligand conformation, and a cis-favored MM-GBSA tendency in the rat-centered MD workflow. In addition, L-RMSF analysis identified atom-wise flexibility differences, whereas the PL-contact analyses described the residue environments sampled by those ligand regions. In difenacoum, the trans isomer showed reduced persistence of the interaction network, particularly around the Phe63/Phe87-centered hydrophobic region and the coumarin oxygen-associated polar-contact region. Taken together, these observations suggest that the trans isomer may possess structural features that render it more prone to dissociation from the VKOR binding site. These results are therefore interpreted as network-level differences in interaction persistence rather than as a strict one-to-one assignment of each ΔRMSF peak atom to a single residue. Such reduced binding stability could, in turn, be associated with a greater likelihood of transition toward metabolic degradation or elimination processes in vivo. In particular, reduced interaction stability may increase the frequency of ligand dissociation from the VKOR binding pocket. Once dissociated, the ligand may become more accessible to hepatic metabolic enzymes, thereby facilitating metabolic degradation and subsequent elimination. In contrast, for flocoumafen, decreased hepatic retention was observed for the cis isomer. In silico analyses revealed that the cis isomer exhibited a restricted range of motion along the binding axis, as indicated by L-torsion analysis. This limitation in torsional flexibility may impose stronger steric constraints on the ligand, thereby hindering conformational reorientation within the binding pocket and potentially compromising binding stability. Accordingly, the preferential elimination observed for the cis isomer may be associated with differences in ligand conformational flexibility rather than interaction strength alone. However, comparison across compounds also indicated clear cases of discordance. For brodifacoum, docking and IC_50_ favored the cis isomer whereas MM-GBSA showed a trans-favored tendency; for difethialone, computational metrics were broadly cis-favored whereas the in vitro VKOR inhibition data showed no clear cis/trans difference; and for bromadiolone, the computational and experimental results remained mixed, with no single metric adequately explaining the observed behavior (Table S5). Collectively, these findings suggest that isomer-dependent differences in binding stability—and the structural factors that influence it, including interaction persistence and conformational flexibility—may contribute to compound-specific hepatic retention patterns. While such associations do not establish direct causality, they provide a mechanistic framework that may help contextualize isomer-selective pharmacokinetic behavior observed in vivo and in wildlife monitoring studies. The hepatic residue analysis in the present study should be interpreted as a supportive within-compound time-course analysis rather than as a standardized pharmacokinetic comparison across different SGARs. This is because the administered cis/trans compositions differed among compounds, and pure isolated isomers were not uniformly available for all SGARs. Accordingly, differences in the initial isomeric composition may have influenced the observed temporal residue profiles and may limit direct quantitative comparisons between compounds. In the present study, the hepatic dataset was therefore used primarily to identify relative cis/trans residue tendencies within each compound. Future studies using standardized administration ratios or purified individual isomers will be necessary for more rigorous cross-compound pharmacokinetic evaluation. In addition, the hepatic isomer quantification in this study was performed by HPLC with UV detection, and the available analytical support consisted of chromatographic separation, representative chromatograms (Supplementary Figure S7), retention-time information, chromatographic resolution values, and calibration data (Supplementary Table S4). Although these data support chromatographic peak assignment and isomer separation under the present conditions, full analytical validation parameters such as LOD/LOQ and repeatability/precision were not comprehensively established in the present study and should therefore be considered a methodological limitation.

L-torsion analysis further indicated that flocoumafen and bromadiolone possess more than twice the number of rotatable bonds compared with the other SGARs examined, suggesting that increased conformational diversity may influence their binding behavior. Such enhanced internal conformational flexibility could allow these ligands to sample a broader range of orientations within the VKOR binding pocket, potentially affecting both interaction patterns and apparent binding stability. For bromadiolone, L-RMSF analysis showed that interaction fractions with VKOR residues corresponding to atoms exhibiting larger cis/trans fluctuations (Ser117, Thr138, and Asn142) were higher for the cis isomer, whereas RMSD analysis indicated greater overall stability for the trans isomer. These observations suggest that the frequency with which interactions occur does not necessarily correspond to the extent to which such interactions are maintained over time. L-torsion analysis further demonstrated that the trans isomer possesses greater internal conformational flexibility, allowing flexible reorientation of the aromatic moieties. Such internal conformational flexibility may enable the trans isomer to adapt to gradual changes in binding mode while maintaining ligand–VKOR interactions, thereby contributing to the lower RMSD values observed. Consistent with this interpretation, previous molecular dynamics studies have highlighted the contribution of ligand flexibility and internal conformational flexibility to apparent binding stability [41,42,43]. In contrast, in vitro VKOR inhibition assays revealed a significant isomeric difference only for flocoumafen, with the cis isomer exhibiting a lower IC_50_ value, a trend opposite to that observed for hepatic retention in vivo. However, VKOR inhibition potency reflects pharmacodynamic activity, whereas hepatic retention reflects pharmacokinetic behavior, and these two properties do not necessarily coincide. In particular, the HEK293T cell-based VKOR assay used in this study primarily reflects inhibitory capacity under relatively static conditions and may not fully capture in vivo processes relevant to hepatic retention, such as ligand dissociation kinetics, metabolic enzyme accessibility, and tissue distribution in Rattus norvegicus. Therefore, stronger VKOR inhibition does not necessarily translate into prolonged hepatic residence time. Taken together, consideration of both concordant trends across multiple compounds and discordant observations such as those seen in vitro suggests that isomer-dependent hepatic retention of SGARs may be associated with differences in ligand flexibility and the stability of interactions within the VKOR binding site, rather than with a single dominant determinant. Consistent with this interpretation, the raw SID outputs indicated that the higher-fluctuation regions of flocoumafen and bromadiolone were embedded in mixed interaction networks that included both hydrophobic contacts and polar or water-mediated contacts, rather than a single dominant hydrogen bond alone. In flocoumafen, Tyr139/Cys135 and Phe63 remained prominent in the filtered contact schematics, while Ser113 provided an additional polar contact near the ether-containing side chain. In bromadiolone, Tyr139/Cys135, Phe63, and Ser117/Thr138-associated contacts coexisted depending on the isomer. These observations suggest that ether oxygens may function as auxiliary hydrogen-bond acceptors. Ether oxygens are generally recognized as secondary hydrogen-bond acceptors [44,45], and in the present analyses, the ether oxygen is best interpreted as one contributor to local polar-contact geometry rather than as the sole structural explanation for the fluctuation differences. Importantly, Tyr139 is a biologically well-established resistance-associated VKOR residue; therefore, the absence or weaker appearance of Tyr139 in some filtered contact schematics should not be interpreted as evidence against its biological relevance, but rather as a trajectory- and representation-dependent outcome. In contrast, for the cis isomer, the corresponding hydrogen bonds were weaker, which may hinder binding rearrangement and reduce apparent interaction persistence. Overall, the observed isomer-dependent differences in hepatic retention among SGARs may be associated with a combination of ligand flexibility, atomic-level fluctuations, interaction stability within the VKOR binding pocket, and structural adaptability mediated by ether oxygen-assisted hydrogen bonding. Although these interpretations remain correlative, the present study, for the first time in SGARs, highlights the potential importance of ligand-intrinsic atomic flexibility and the role of ether oxygen as an auxiliary hydrogen-bond acceptor as structural parameters to consider in the design and evaluation of SGAR isomers. Although experimentally resolved human VKOR structures provide important references for inhibitor-bound conformations, AF2-derived VKOR models were retained as the primary comparative framework in this study to maintain consistency across multiple species and with the rodent-centered biological design of the work. Because available ligand-bound VKOR crystal structures are currently limited mainly to human VKOR and to a small number of ligands, their exclusive use would not allow uniform cross-species comparison. Supplementary docking using the available human VKOR crystal structures showed broadly similar cis/trans tendencies for several compounds, supporting the utility of the AF2-based framework for comparative structural analysis, while also indicating compound-dependent structure sensitivity. Notably, the available experimental VKOR–SGAR complex corresponds to cis-brodifacoum, which is compatible with the possibility that a cis-configured SGAR can adopt a stable inhibitor-bound conformation in VKOR, although this should not be generalized to all SGARs.

In the present study, isomer-specific binding modes and relative stability were evaluated using in silico approaches, including molecular docking scores, RMSD, L-RMSF, and torsional analyses. While these metrics provide useful insights into ligand–VKOR interaction behavior, they do not fully capture long-term target engagement, downstream metabolic enzyme activity, or dynamic elimination processes. Accordingly, the quantitative reproduction of in vivo behavior based solely on these computational indices remains inherently limited. In addition, the VKOR structure employed in this study was derived from *Rattus norvegicus*. Because interspecies differences in VKORC1 are likely to influence isomer-specific binding and are directly relevant to mitigating secondary poisoning in non-target wildlife, further validation across multiple species is warranted. As VKORC1 sequences and structures vary among species, such structural differences may further modulate isomer-selective ligand binding and retention patterns across different animals. In particular, species-dependent VKOR variability could influence SGAR retention patterns in non-target wildlife exposed through secondary poisoning. Another unresolved aspect concerns the extent to which cis–trans interconversion of the isomers themselves—via enzymatic or non-enzymatic pathways—may occur in vivo. Addressing this question through in vitro liver microsome systems represents an important next step toward clarifying the contribution of isomer metabolism to observed residue patterns. Notably, preliminary in silico ADMET Predictor analyses did not reveal marked cis/trans differences in predicted physicochemical or ADMET-related descriptors (Table S3). This result suggests that the observed isomer-dependent residue patterns are unlikely to be explained solely by coarse-grained predicted properties such as lipophilicity, solubility, permeability, or general metabolic liability. At the same time, such descriptor-based prediction tools are not necessarily optimized to detect subtle stereochemical effects arising from cis–trans isomerism, particularly when the biological outcome may depend on local conformational behavior, target dissociation, membrane-associated orientation, or stereoselective metabolic recognition. Therefore, the absence of marked cis/trans differences in these in silico predictions should not be interpreted as evidence against isomer-dependent pharmacokinetic behavior, but rather as an indication that higher-resolution experimental and structural analyses will be required. Experimental validation using liver microsomal systems will therefore be necessary to determine whether metabolic processes contribute to the observed isomer-dependent residue patterns. Despite these limitations, the present study supports the view that intrinsic differences in ligand molecular structure may contribute, in some compounds, to cis/trans-dependent hepatic retention trends that are broadly compatible with existing field monitoring observations. By focusing on cis–trans isomer-specific binding behavior at the molecular level, this work provides a mechanistic framework for interpreting how differences in ligand–VKOR interactions may contribute to isomer-selective hepatic retention, while also highlighting that such relationships are compound-dependent and not uniformly concordant across all SGARs. The present results suggest, across multiple compounds, that structural parameters such as ligand flexibility and internal conformational flexibility may influence binding stability and, consequently, hepatic retention, highlighting their relevance as factors to consider in SGAR isomer design and evaluation. These findings further suggest that consideration of isomer selection or isomer-enriched formulation strategies may represent a potential approach for designing rodenticides with reduced ecological risk while maintaining efficacy against target rodents.

## 4. Materials and Methods

### 4.1. Analyzed Compounds and Isomeric Structures

The second-generation anticoagulant rodenticides analyzed in this study—difenacoum, brodifacoum, difethialone, flocoumafen, and bromadiolone—were examined in their respective cis and trans isomeric forms (Table 2). All compounds share a coumarin core structure and possess one or more chiral centers, indicated by asterisks (*), which give rise to cis/trans stereoisomerism. Using these defined isomeric structures, we conducted an integrated evaluation across multiple experimental and computational layers, including hepatic retention studies, factor IX (FIX) activity inhibition assays, and in silico analyses comprising molecular docking and molecular dynamics simulations.

### 4.2. Materials and Reagents

Ultrapure water, ethanol, methanol, Dulbecco’s Modified Eagle’s Medium (DMEM; high glucose, supplemented with L-glutamine, phenol red, and sodium pyruvate), penicillin–streptomycin solution (100×), phosphate-buffered saline without calcium and magnesium (PBS(−)), hygromycin B solution (50 mg/mL), dimethyl sulfoxide (DMSO), and diphacinone standard were purchased from FUJIFILM Wako Pure Chemical Corporation (Osaka, Osaka, Japan). Second-generation anticoagulant rodenticides (brodifacoum, difethialone, flocoumafen, bromadiolone, and difenacoum) used for exposure experiments were synthesized by Daimaru Compound Chemical Co., Ltd. (Nagano, Nagano, Japan). Heat-inactivated fetal bovine serum was obtained from Cytiva (Okubo, Tokyo, Japan). Nuclease-free water was purchased from Life Technologies (Waltham, MA, US). Revohem FIX synthetic substrate and human standard plasma were purchased from Sysmex Corporation (Kobe, Hyogo, Japan). The coding sequence of *Rattus norvegicus* vitamin K epoxide reductase (VKOR; GenBank accession no. NM_203335.2) was synthesized by GenScript (Chiyoda, Tokyo, Japan) and cloned into the pcDNA3.1 expression vector. TransIT-293 2020 transfection reagent was purchased from Takara Bio Inc (Kusatsu, Shiga, Japan). Vitamin K epoxide, Opti-MEM reduced-serum medium, warfarin, and a Multiskan FC absorbance microplate reader were purchased from Thermo Fisher Scientific (Minato, Tokyo, Japan). HEK293T cells were provided by RIKEN (Wako, Saitama, Japan). Collagen I-coated culture dishes (60 mm) and 24-well multiwell plates were purchased from AGC Techno Glass (Yoshida, Shizuoka, Japan). A mini portable CO_2_ incubator was obtained from AS ONE Corporation (Osaka, Osaka, Japan). Two-milliliter and 1.5-milliliter microcentrifuge tubes were purchased from Fukae Kasei Group (Kobe, Hyogo, Japan), and 15 mL sterile centrifuge tubes were obtained from Sansei Medical Instruments (Chiyoda, Tokyo, Japan).

### 4.3. Hepatic Retention Study

Five second-generation anticoagulant rodenticides—difenacoum, brodifacoum, difethialone, flocoumafen, and bromadiolone—were used as test compounds. Each compound was prepared as a cis/trans isomeric mixture, with isomer ratios of 1:1 (difenacoum), 6:4 (brodifacoum), 1:1 (difethialone), 2:8 (flocoumafen), and 8:2 (bromadiolone). Male Wistar rats (7 weeks old) were orally administered each rodenticide mixture at a dose of 0.1 mg/kg body weight. Liver tissues were collected at 1, 4, 7, and 14 days after administration (*n* = 2 per time point). Hepatic concentrations of the cis and trans isomers were quantified by high-performance liquid chromatography (HPLC) with UV detection. Cis and trans isomers were chromatographically resolved and quantified separately, and hepatic retention was expressed as a relative value, with the concentration measured on day 1 post-administration defined as 100%. Quantitative analysis of rodenticides was performed using a high-performance liquid chromatography (HPLC) system (Prominence, Shimadzu, Japan) equipped with a UV absorbance detector. Chromatographic separation was carried out using a C18 ODS column under isocratic conditions. The mobile phase consisted of acetonitrile (MeCN), tetrahydrofuran (THF), trifluoroacetic acid (TFA), and water (H_2_O). The solvent compositions (*v*/*v*) were as follows: difenacoum, MeCN/THF/TFA/H_2_O = 54.945:3.375:0.1:41.58; brodifacoum, 59.94:3:0.1:36.96; difethialone, 69.93:2.25:0.1:27.72; flocoumafen, 59.94:3:0.1:36.96; and bromadiolone, 49.95:3.75:0.1:46.2. UV detection was performed at 254 nm. Representative chromatograms of cis and trans isomers and an example chromatogram of a liver extract sample are shown in Supplementary Figure S7. Retention-time data, chromatographic resolution values, and calibration information are summarized in Supplementary Table S4.

### 4.4. In Vitro Inhibition of FIX Activity by Anticoagulant Rodenticides

An in vitro factor IX (FIX) activity inhibition assay was performed using HEK293T cells, based on the method originally described by Fregin et al. [46] and subsequently modified by Sato and Watanabe et al. [47]. In this study, a human FIX–stably expressing HEK293T cell line previously established by Sato et al. [47] was used. Cells were maintained on collagen-coated dishes in Dulbecco’s Modified Eagle’s Medium (DMEM; high glucose) supplemented with 10% fetal bovine serum (FBS) and 1% penicillin–streptomycin at 37 °C under 7.5% CO_2_. After routine passaging and selection with hygromycin B, cells were seeded into 24-well plates at approximately 90% confluence (1.0 × 10^5^ cells/mL, 1 mL per well) and cultured for 24 h prior to experimentation. For rodenticide exposure experiments, cells in each well were transfected with a *Rattus norvegicus* VKOR expression plasmid using TransIT-293 transfection reagent. Following transfection, the culture medium was replaced with Opti-MEM supplemented with charcoal-treated and heat-inactivated FBS. Vitamin K epoxide (VKO; ethanol/methanol = 1:1) was added to the medium at a final concentration of 10 µM by a 1:1000 dilution of a 10 mM stock solution, resulting in a final organic solvent concentration of 0.1%. Subsequently, cis and trans isomers of anticoagulant rodenticides were added at designated concentrations. First-generation rodenticides (warfarin and diphacinone) were tested at final concentrations of 0, 5, 10, 25, 100, and 250 nM, whereas second-generation rodenticides (difenacoum, brodifacoum, difethialone, flocoumafen, and bromadiolone) were evaluated at 0, 2.5, 5, 10, 25, and 100 nM. After 72 h of incubation, culture supernatants (500 µL per well) were collected and stored at −20 °C until analysis. FIX activity in the supernatants was determined using a synthetic substrate-based chromogenic assay (Revohem FIX synthetic substrate assay; Sysmex Corporation, Kobe, Hyogo, Japan) according to the manufacturer’s instructions. Briefly, 50 µL of diluted supernatant was mixed with 50 µL each of FIX R1 (human factors VIII and X reagent), FIX R2 (activator), and FIX R3 (substrate reagent), followed by incubation at 37 °C. Absorbance at 450 nm was measured using a microplate reader (Multiskan FC; Thermo Fisher Scientific, Minato, Tokyo, Japan). A standard curve was generated using human standard plasma with a dilution series ranging from 0- to 1500-fold. Each condition was measured in quadruplicate within a single experiment. Reproducibility of the concentration–response trend was assessed in independent experiments using difenacoum as a representative compound. In preliminary experiments, both cis and trans isomers of difenacoum exhibited near-complete inhibition of FIX activity at 100 nM (Supplementary Figure S1). Based on this observation, second-generation rodenticides, including difenacoum, were evaluated within a concentration range of 0–100 nM in the main experiments.

### 4.5. In Silico Molecular Docking of Rodenticides to VKOR

The predicted binding poses and relative docking score tendencies between anticoagulant rodenticides and vitamin K epoxide reductase (VKOR) were comparatively evaluated using in silico molecular docking. Three-dimensional ligand structures were obtained as SDF files from PubChem (S-warfarin: CID 54688261; diphacinone: CID 6719; second-generation rodenticides are listed in Table 2). Three-dimensional VKOR structures from four species (*Rattus norvegicus*, *Rattus rattus*, *Mus musculus*, and *Homo sapiens*) were obtained as mmCIF files from the AlphaFold Protein Structure Database and used as a unified structural framework for comparative docking analysis. We selected AF2-derived structures as the primary platform because the present study aimed to compare ligand-binding behavior across multiple species under identical computational conditions, while maintaining consistency with the rodent-centered biological context of the study. In particular, the in vivo residue analysis focused on rats, and the mechanistic interpretation was anchored to the rodent VKOR system. Thus, the cross-species docking analysis was intended as an exploratory comparative assessment of ligand-binding tendencies rather than as a direct quantitative measure of binding free energy. RMSD values were recorded both after automatic exclusion of poorly fitting matched atom pairs and without this exclusion. The subsequent molecular dynamics simulations were not initiated directly from the cross-species rigid-docking poses, but from induced-fit-refined complexes based on the closed-state R. norvegicus VKOR model, as described below.

To additionally assess the structural plausibility of the AF2-based framework, supplementary docking analyses were also performed using available experimentally resolved human VKOR structures (PDB IDs: 6WV3 and 6WVH), and the resulting cis/trans docking tendencies were compared with those obtained from the AF2-based models. In addition, the docking-ready closed-state AF2-derived human VKOR model was superposed on the experimentally resolved closed-state human VKOR structure using UCSF ChimeraX Matchmaker. The corresponding UniProt accession numbers were Q6TEK4 (*R. norvegicus*), I4DXG3 (*R. rattus*), Q9CRC0 (*M. musculus*), and Q9BQB6 (*H. sapiens*). VKOR adopts two redox-dependent conformational states—open and closed—defined by the formation of disulfide bonds among four catalytically relevant cysteine residues. In the open state, disulfide bonds are formed between Cys43–Cys51 and Cys132–Cys135, whereas in the closed state, a disulfide bond is formed between Cys51–Cys132. For each AlphaFold-predicted VKOR structure, both open and closed conformations were individually generated by manually editing the corresponding disulfide bond patterns using Maestro (Schrödinger), followed by structural optimization via energy minimization with the OPLS4 force field.

Molecular docking was performed using Glide (Schrödinger Release 2023; Schrödinger) in standard precision (SP) mode. For each AF2-based VKOR model, the docking grid was defined by superimposing the experimentally resolved human VKORC1 structure (PDB ID: 6WV3) onto the target model and using the aligned warfarin coordinates as a reference for the canonical inhibitor-binding pocket, since this site corresponds to the experimentally observed antagonist-binding region relevant to competitive VKOR inhibition. Docking scores (Glide Score) were used as comparative indices for pose ranking within the predefined VKOR binding pocket under identical docking conditions and were not interpreted as direct quantitative measures of binding free energy. For each ligand–VKOR pair, the lowest-energy (best-scoring) docking pose was selected for subsequent analyses. To assess the structural plausibility of the most stable docking pose, the selected pose was subjected to energy minimization and used as the reference conformation for root mean square deviation (RMSD) analysis (Figure 4b). RMSD values were calculated to quantify structural deviations of the docked ligand relative to this energy-minimized reference structure. Smaller RMSD values were interpreted as indicating higher conformational stability of the docking pose. Docking simulations were conducted under identical conditions for all four VKOR species to enable direct comparison of relative docking score tendencies and docking-pose characteristics across species.

### 4.6. In Silico Molecular Dynamics Simulations

#### 4.6.1. Production Run

Molecular dynamics (MD) simulations were performed using Desmond (Schrödinger Release 2025-2; Schrödinger, New York, NY, USA) with the open-state VKOR structure in complex with each second-generation anticoagulant rodenticide, using the optimal docking pose as the initial structure based on our previous report [48]. The open conformation was selected because it is considered to more reliably reproduce ligand-protein interaction stability under physiological conditions [49]. The system was embedded in a POPC lipid bilayer and solvated using the TIP3P water model. Sodium and chloride ions were added to achieve a physiological ionic strength of 0.15 M. The OPLS4 force field was applied to all components of the system. Production MD simulations were conducted for 100 ns. Trajectories were recorded at a total of 2500 frames, and all other simulation parameters were set to their default values in Desmond.

#### 4.6.2. Ligand Root Mean Square Fluctuation (L-RMSF) Analysis

Ligand root means square fluctuation (L-RMSF), which quantifies atom-wise ligand flexibility during MD simulations, was calculated from the 100 ns trajectories (2500 frames) using the Simulation Interactions Diagram (SID) module. L-RMSF values were compared between cis and trans isomers for each compound. Higher L-RMSF values reflect greater ligand flexibility and reduced interaction stability. To directly assess isomer-dependent differences in ligand flexibility, the difference between isomers was defined as

ΔRMSF = L-RMSF_trans − L-RMSF_cis(1)

Positive ΔRMSF values indicate higher flexibility of the trans isomer, whereas negative values indicate higher flexibility of the cis isomer.

#### 4.6.3. Protein–Ligand Contact (PL-Contact) Analysis

Protein–ligand interactions were evaluated using the SID module (Simulation Interaction Diagram). For each trajectory frame (2500 frames), hydrogen bonds, hydrophobic contacts, π–π interactions, and water bridges were extracted from the corresponding output files (e.g., PL-Contacts_HBond.dat). For each ligand–residue pair, the number of frames in which an interaction was observed was counted, and the interaction fraction was calculated as the ratio of interaction-positive frames to the total number of frames. Two complementary SID outputs were used in the present study: (i) the residue-wise Protein–Ligand Contacts summary/timeline outputs, which were used for residue-level interaction fraction analyses; and (ii) the atom-wise Ligand–Protein Contacts schematics, which display only interactions occurring for >5% of the simulation time and were used for visual localization of contacting ligand atoms. Accordingly, residue-level interaction fractions and atom-wise contact schematics were interpreted as complementary, not strictly one-to-one equivalent, representations.

#### 4.6.4. Ligand Torsion (L-Torsion) Analysis

Time-dependent dihedral angle variations in rotatable ligand bonds were extracted using the SID module. Angular distributions and their standard deviations were calculated for each rotatable bond and compared as measures of internal conformational flexibility. A larger standard deviation indicates a higher degree of internal conformational flexibility around the corresponding bond axis, reflecting increased internal conformational flexibility of the ligand.

### 4.7. In Silico ADMET Prediction

Physicochemical and metabolic properties of the SGAR isomers were predicted using ADMET Predictor™ 13.0 (Simulations Plus, Inc., Lancaster, CA, USA). The predicted parameters included physicochemical descriptors (logP, logD, solubility, PSA, and rotatable bonds) and CYP-mediated metabolic properties, as summarized in Supplementary Table S3.

### 4.8. Statistical Analysis

#### 4.8.1. IC_50_ Analysis

VKOR activity was quantified based on absorbance measurements and normalized by defining the activity in the absence of inhibitors as 100%. Concentration–response curves were fitted using a nonlinear regression model, and IC_50_ values were calculated using GraphPad Prism (version 9). For multiple comparisons, *p*-values were adjusted using a false discovery rate (FDR) correction. Statistical significance was assessed based on the FDR-adjusted *p*-values.

#### 4.8.2. Molecular Docking and Molecular Dynamics Analyses

Normality of each dataset was evaluated using the Shapiro–Wilk test. When the data were normally distributed, comparisons between cis and trans isomers were performed using paired *t*-tests, as the isomeric data were derived from the same compounds. When normality was not satisfied, the nonparametric Wilcoxon signed-rank test was applied. In the present study, the Wilcoxon signed-rank test was used for docking score comparisons of bromadiolone and for RMSD comparisons of difethialone and flocoumafen.

## 5. Conclusions

Taken together, the findings of this study indicate that differences in hepatic retention between cis and trans isomers of five major SGARs were associated with variations in ligand flexibility, atomic-level fluctuations, and interaction patterns within the VKOR binding site. Through a systematic in silico analysis encompassing all five compounds, compound-dependent structural hypotheses—particularly ligand flexibility, local conformational adaptability, and the balance between hydrophobic and polar contact networks—were identified as candidate factors associated with isomer-dependent retention behavior. Such structure-oriented insights are informative at early stages of compound evaluation, prior to in vivo testing, by providing qualitative indicators for anticipating residue persistence and selectivity. In this context, in silico analyses support the selection or refinement of cis–trans isomer compositions and chemical features that maintain efficacy in target species while reducing the likelihood of persistence and accumulation in non-target wildlife. Future integration of VKOR structural models from multiple species with metabolic pathway information in hybrid in silico–in vitro frameworks may support the development of predictive safety assessment models based on molecular structural characteristics.

## Figures and Tables

**Table 1:** IC_50_ values determined from concentration–response inhibition curves.

Compound	IC_50_ (nM)	
	Cis	Trans
**d** **ifenacoum**	1.4 ± 0.5	3.0 ± 0.6
**b** **rodifacoum**	1.3 ± 0.1	4.1 ± 0.5
**d** **ifethialone**	4.8 ± 0.2	3.8 ± 0.2
**f** **locoumafen**	2.2 ± 0.2	7.5 ± 0.1 *
**b** **romadiolone**	4.4 ± 0.2	2.3 ± 0.1
**s** **-** **w** **arfarin**	21.9 ± 0.3	
**d** **iphacinone**	15.5 ± 0.2	

IC_50_ values are expressed as the mean ± SEM (*n* = 4). An asterisk (*) indicates a statistically significant difference between cis and trans isomers (*p* < 0.05). IC_50_ values represent the inhibitor concentration required to achieve 50% inhibition of Factor IX activity.

**Table 2:** Chemical structures of second-generation anticoagulant rodenticides. Chiral centers are indicated by asterisks (*).

Rodenticides	Structural Formula	Pubchem ID	
Difenacoum	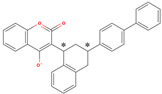	Cis	54739296
		Trans	54707610
Brodifacoum	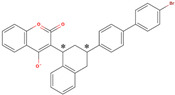	Cis	76962528
		Trans	54716798
Difethialone	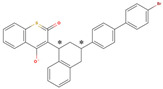	Cis	76965665
		Trans	76965663
Flocoumafen	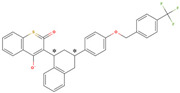	Cis	76960465
		Trans	76960466
Bromadiolone	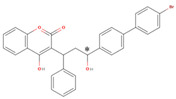	Cis (1S3S)	76956796
		Trans (1S3R)	76956795

## Data Availability

The data supporting the findings of this study are available from the corresponding author upon reasonable request.

## Supplementary Materials

The following supporting information can be downloaded at: https://www.mdpi.com/article/10.3390/ijms27093794/s1.
