# Supplementary material for: Isomer-Dependent Pharmacokinetic Behavior and VKOR Interactions of Second-Generation Anticoagulant Rodenticides: An Integrated In Vivo–In Vitro–In Silico Investigation
Source: Int J Mol Sci. 2026 Apr 24;27(9):3794. doi: 10.3390/ijms27093794 (PMC13164442; doi:10.3390/ijms27093794)
Supplement: Supplementary file 1 [file ijms-27-03794-s001.zip › ijms-4163376-supplementary.pdf]

Isomer-Dependent Pharmacokinetic Behavior and VKOR Interactions of Second-Generation Anticoagulant Rodenticides: An Integrated in vivo–in vitro–in silico Investigation

Authors

Moyu Miyamae[1], Satoru Nagaoka[2], Teppei Hayama[1], Misaki Fukamatsu[1], Ryo Kamata[1], and Kazuki Takeda [1, 3*]

[1] School of Veterinary Medicine, Kitasato University, Towada 034-8628, Japan

[2] Daimaru Compound Chemical Co., Ltd., Nagano 381-1222, Japan

[3] School of Computing, Institute of Science Tokyo, Tokyo 152-8552, Japan

* Correspondence should be addressed to takeda@vmas.kitasato-u.ac.jp (Takeda K).

Supplementary Materials

Figure S1. Preliminary evaluation of FIX activity inhibition by cis and trans isomers of difenacoum.

Figure S2. RMSD comparison of cis and trans isomers of second-generation anticoagulant rodenticides in closed and open VKOR models across four species.

Figure S3. Ligand flexibility analysis of cis and trans isomers of second-generation anticoagulant rodenticides based on L-RMSF analysis.

Figure S4. Schematic representation of protein–ligand interactions for cis and trans isomers of second-generation anticoagulant rodenticides based on PL_Contact analysis.

Figure S5. Distribution of internal ligand torsion angles of second-generation anticoagulant rodenticides in complexes with rat VKOR based on L_torsion analysis.

Figure S6. Protein–ligand RMSD profiles for all rat VKOR–ligand complexes.

Figure S7. Representative HPLC–UV chromatograms supporting cis/trans isomer quantification of second-generation anticoagulant rodenticides (SGARs).

Table S1. Comparison of cis/trans docking score tendencies between AF2-based VKOR models and experimentally resolved human VKOR structures.

Table S2. Comprehensive analysis of protein–ligand interaction fraction for cis and trans isomers of second-generation anticoagulant rodenticides in complexes with rat VKOR based on PL_Contact analysis.

Table S3. In silico ADMET prediction of physicochemical and metabolic parameters for cis and trans isomers of SGAR compounds using ADMET Predictor.

Table S4. Summary of HPLC-UV chromatographic conditions and analytical support for cis/trans isomer quantification.

Table S5. Comparison of computational and experimental cis/trans trends across SGARs.

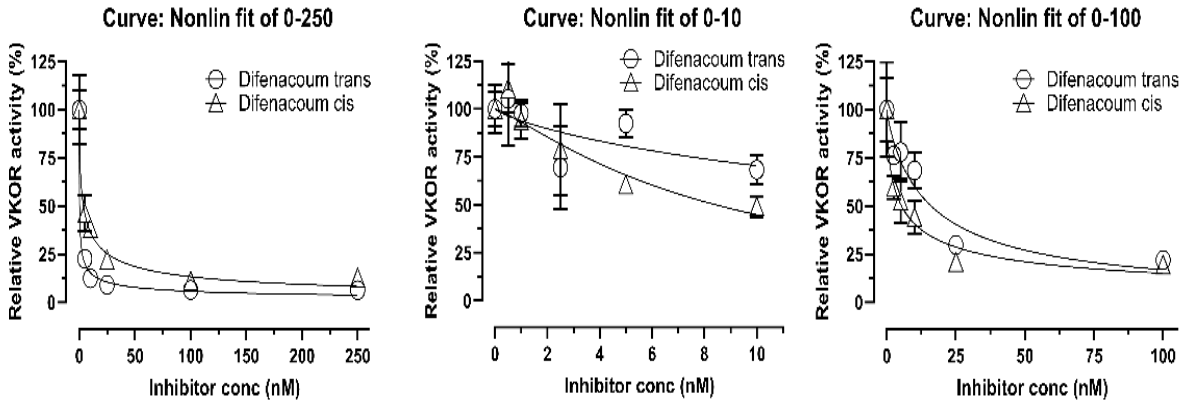

Figure S1. Preliminary evaluation of FIX activity inhibition by cis and trans isomers of difenacoum. FIX activity was assessed in HEK293T cells expressing rat VKOR and factor IX (FIX) following treatment with cis or trans difenacoum at different concentration ranges. Three independent experiments were conducted. In the first experiment (left), final difenacoum concentrations were 0, 5, 10, 25, 100, and 250 nM (n = 4). In the second experiment (middle), concentrations were 0, 0.5, 1, 2.5, 5, and 10 nM (n = 4). In the third experiment (right), concentrations were 0, 2.5, 5, 10, 25, and 100 nM (n = 4). VKOR activity was calculated based on FIX activity and normalized to 100% in the absence of inhibitors. Error bars represent the standard error of the mean (SEM). Triangles indicate cis-difenacoum, and circles indicate trans-difenacoum.

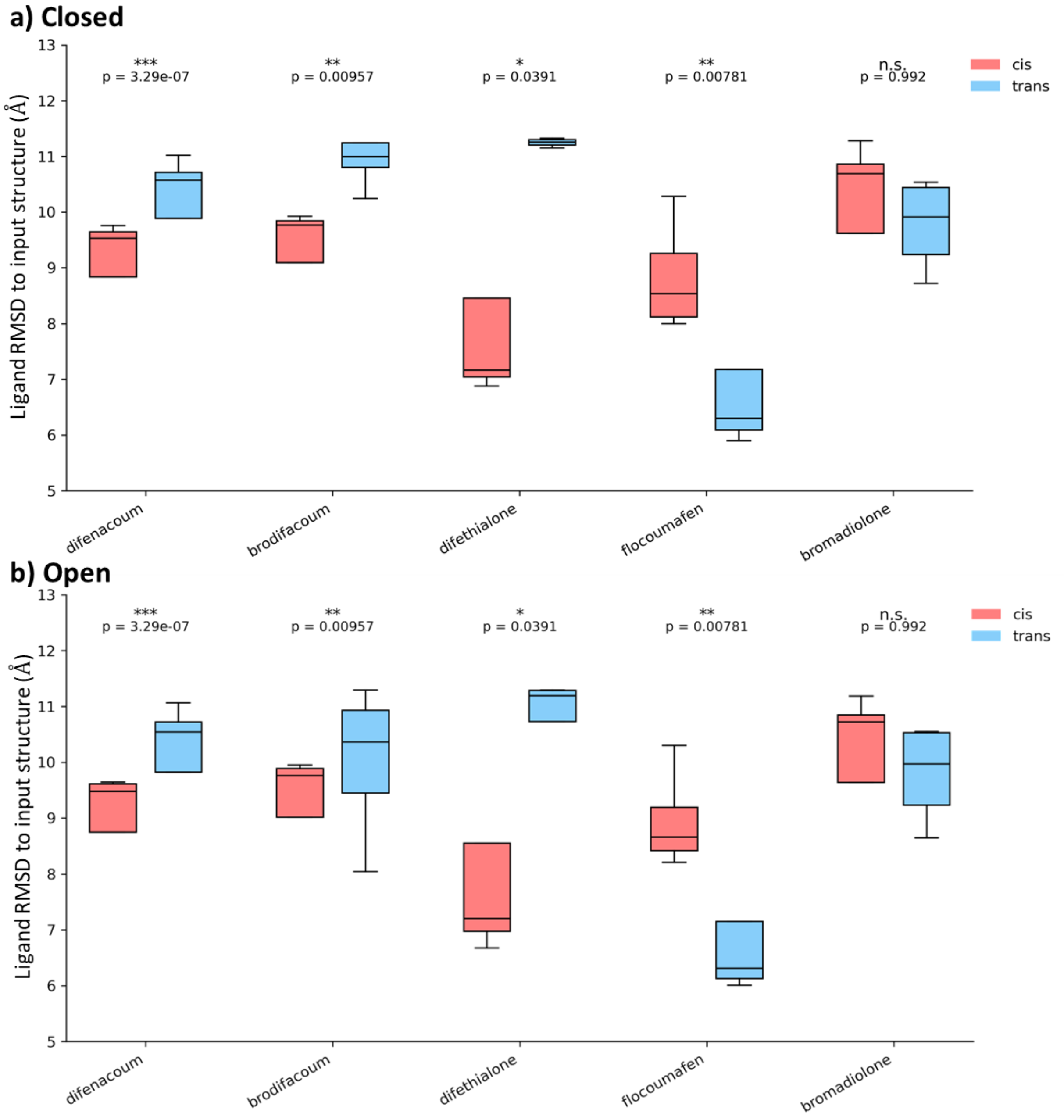

Figure S2. RMSD Comparison of cis and trans Isomers of Second-Generation Anticoagulant Rodenticides in Closed and Open VKOR Models across Four Species. (a) RMSD distributions for cis and trans isomers in the closed-state VKOR model across four species (Rattus norvegicus, Rattus rattus, Mus musculus, and Homo sapiens). (b) RMSD distributions for cis and trans isomers in the open-state VKOR model across the same four species. RMSD values were calculated for ligand atoms relative to the OPLS4-minimized ligand conformations used as the input structures for docking. Lower RMSD values indicate lower structural deviation from the input ligand geometry after docking.

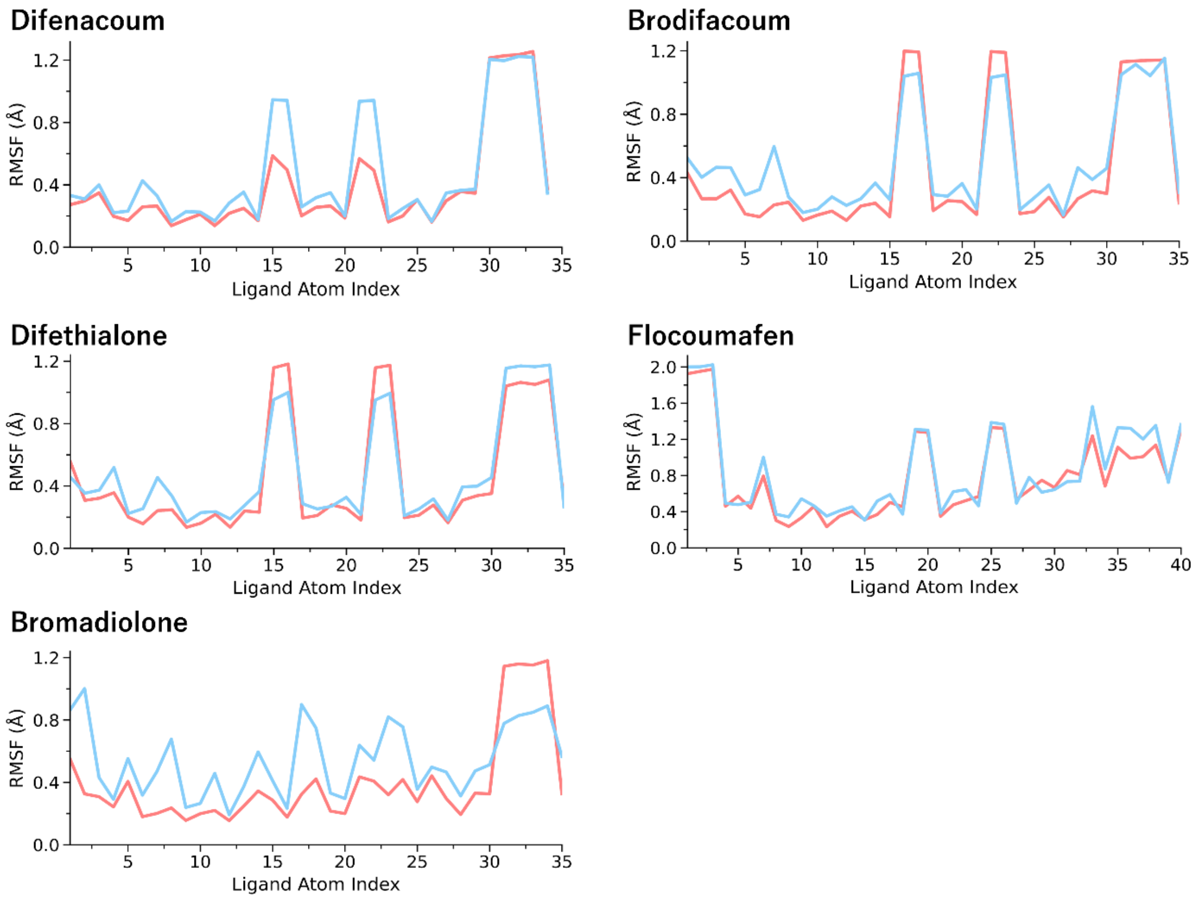

Figure S3. Ligand flexibility analysis of cis and trans isomers of second-generation anticoagulant rodenticides based on L-RMSF analysis. Ligand root mean square fluctuation (L-RMSF) values were calculated for each ligand atom in complexes with rat VKOR. The y-axis represents RMSF values (Å), and the x-axis represents ligand atom indices as defined in the Desmond output. Higher RMSF values indicate greater atomic flexibility. Red lines indicate cis isomers, and blue lines indicate trans isomers.

Difenacoum-cis

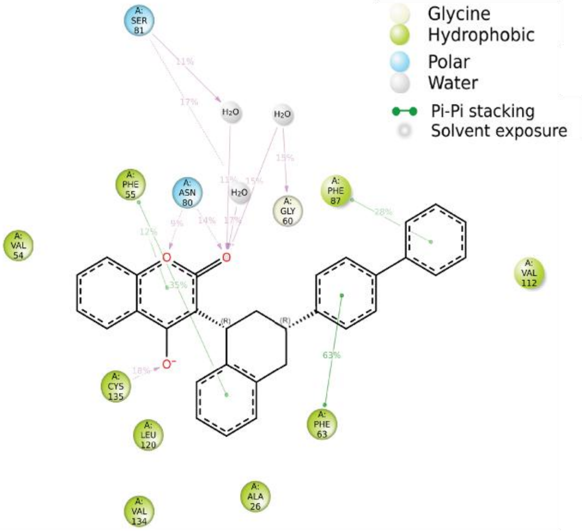

Difenacoum-trans

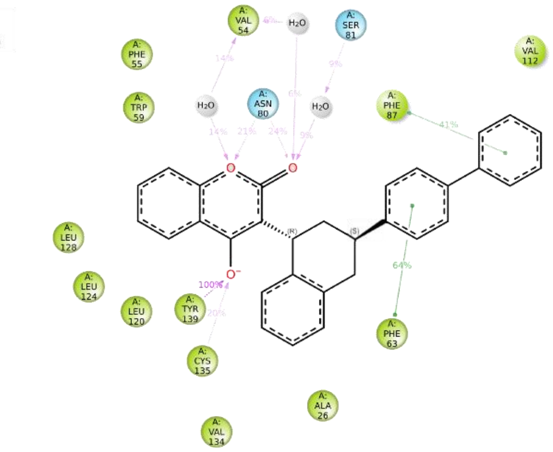

Brodifacoum-cis

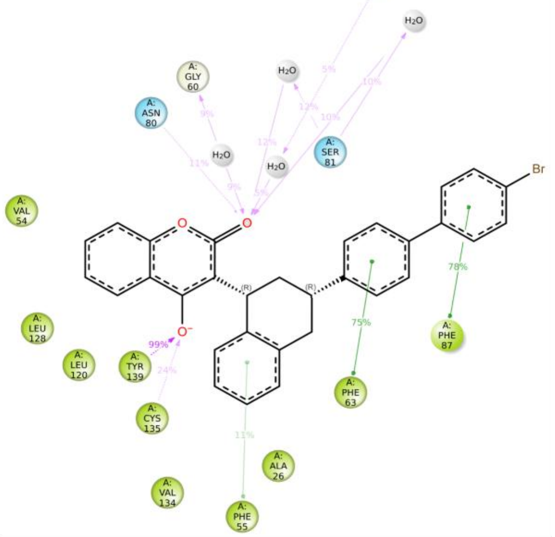

Brodifacoum-trans

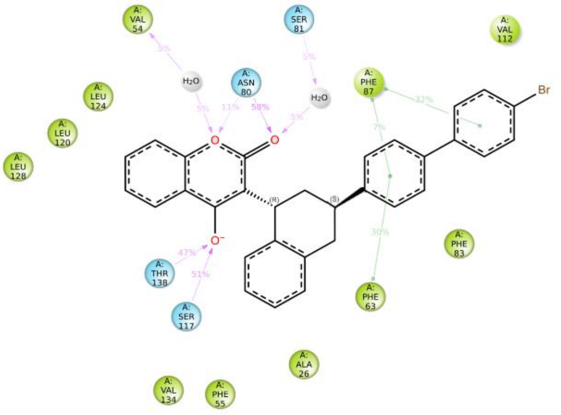

Difethialone-cis

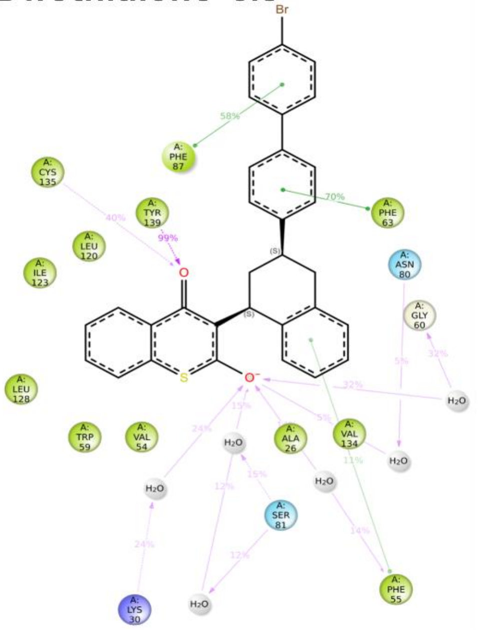

Difethialone-trans

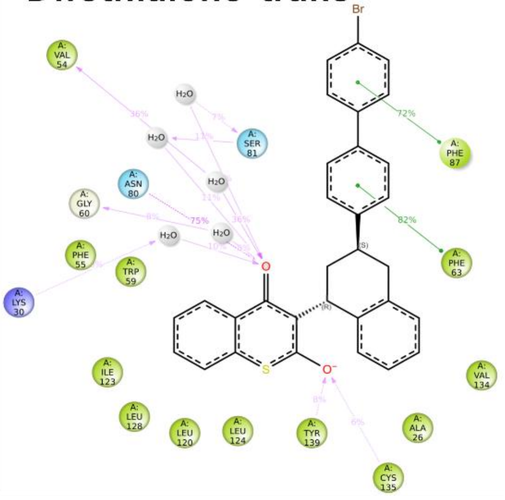

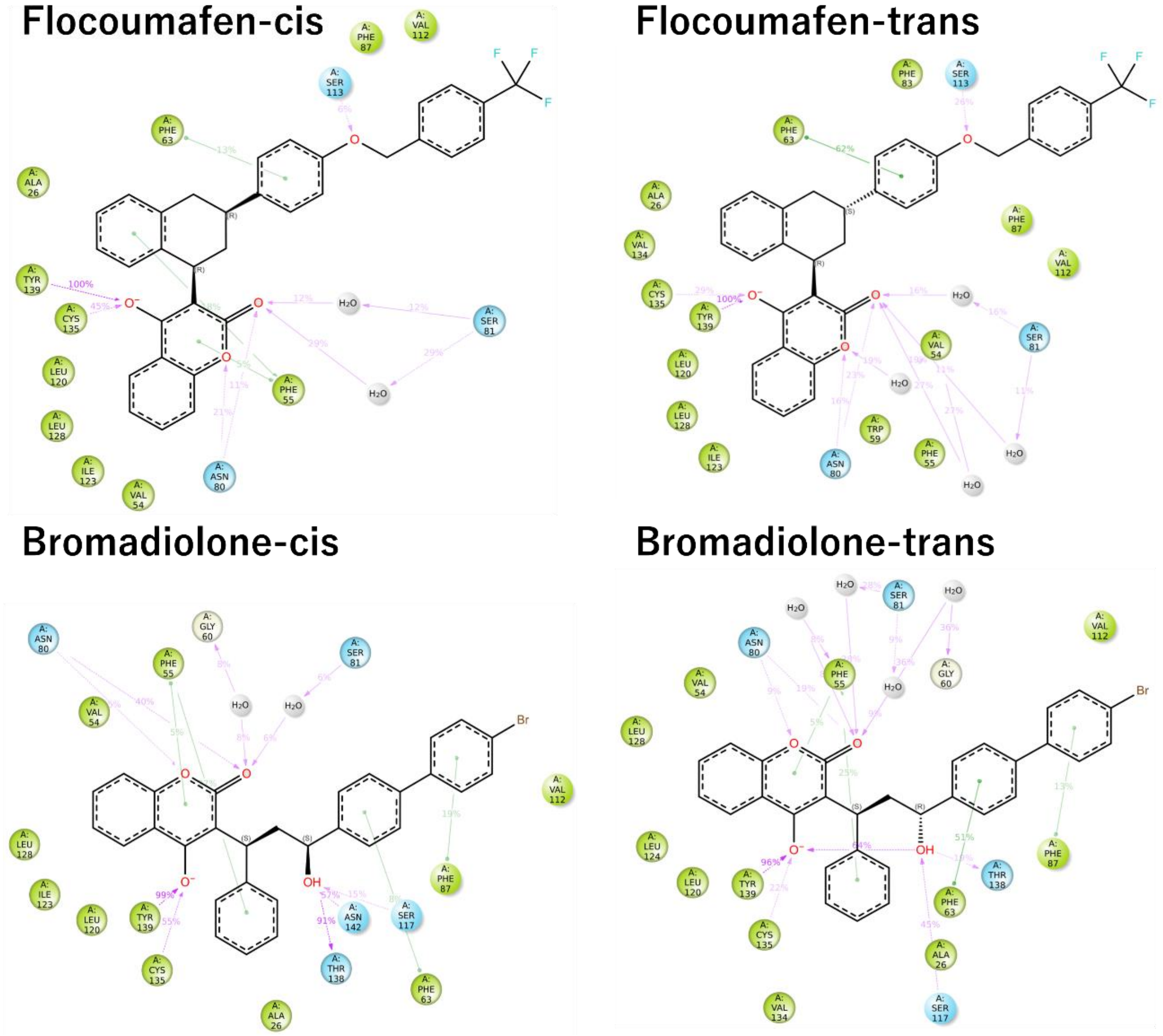

Figure S4. Schematic representation of protein–ligand interactions for cis and trans isomers of second-generation anticoagulant rodenticides based on PL_Contact analysis. Hydrophobic residues are shown in light green, polar residues in light blue, and water-mediated interactions in white. Pink lines indicate non-covalent interactions, and green lines indicate π–π interactions. Numerical values represent the interaction frequency (%) during 100 ns molecular dynamics simulations. For example, a value of 91% indicates that the corresponding interaction was maintained for 91% of the total simulation time.

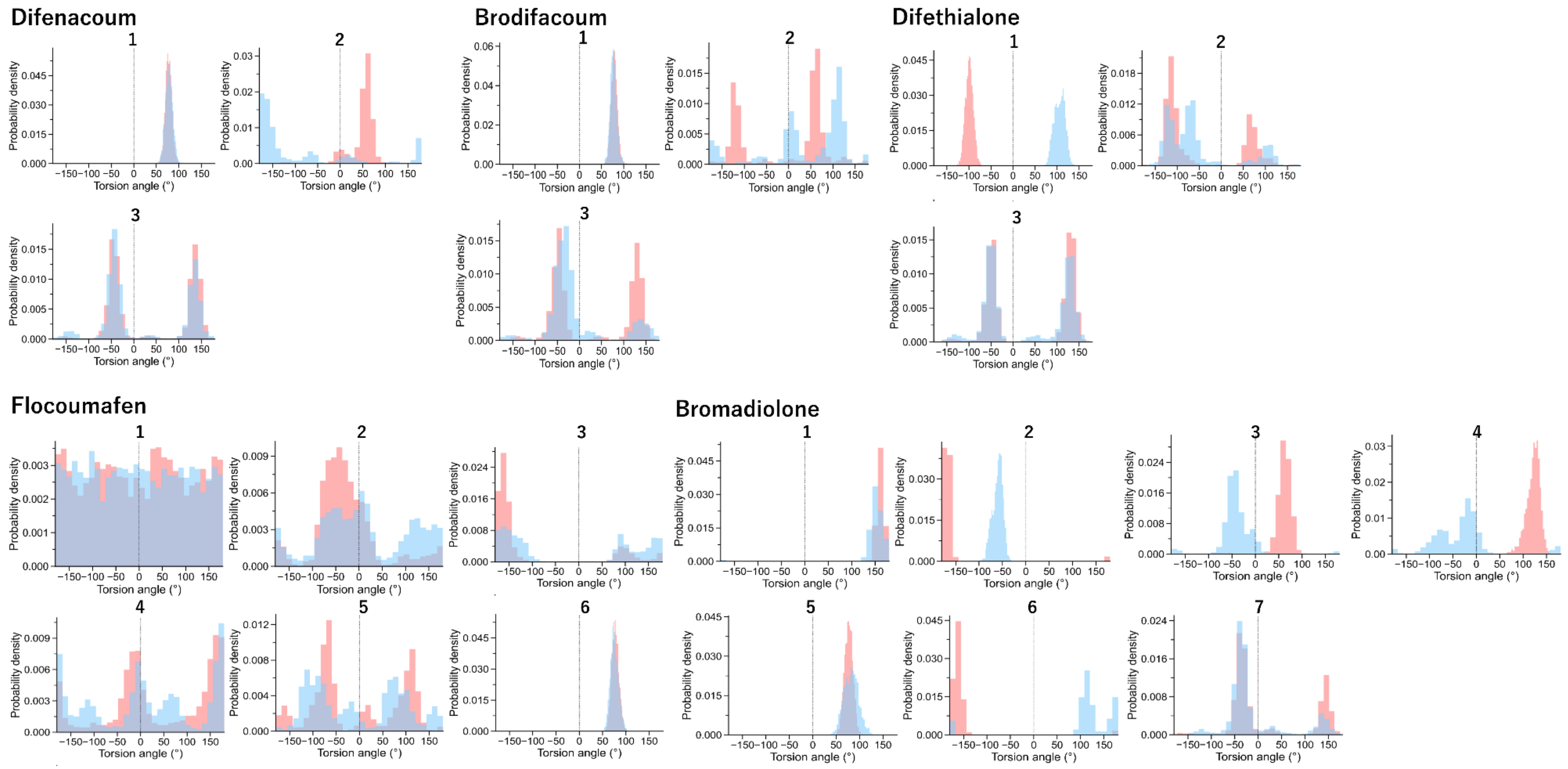

Figure S5. Distribution of internal ligand torsion angles of second-generation anticoagulant rodenticides in complexes with rat VKOR based on L-torsion analysis. Distributions of dihedral angles for each predefined rotatable bond (Torsion1–7) are shown for the cis and trans isomers of each compound over the 100 ns MD simulations. These plots complement the standard deviation summary shown in Figure 7 by illustrating the range of conformations sampled around each bond axis.

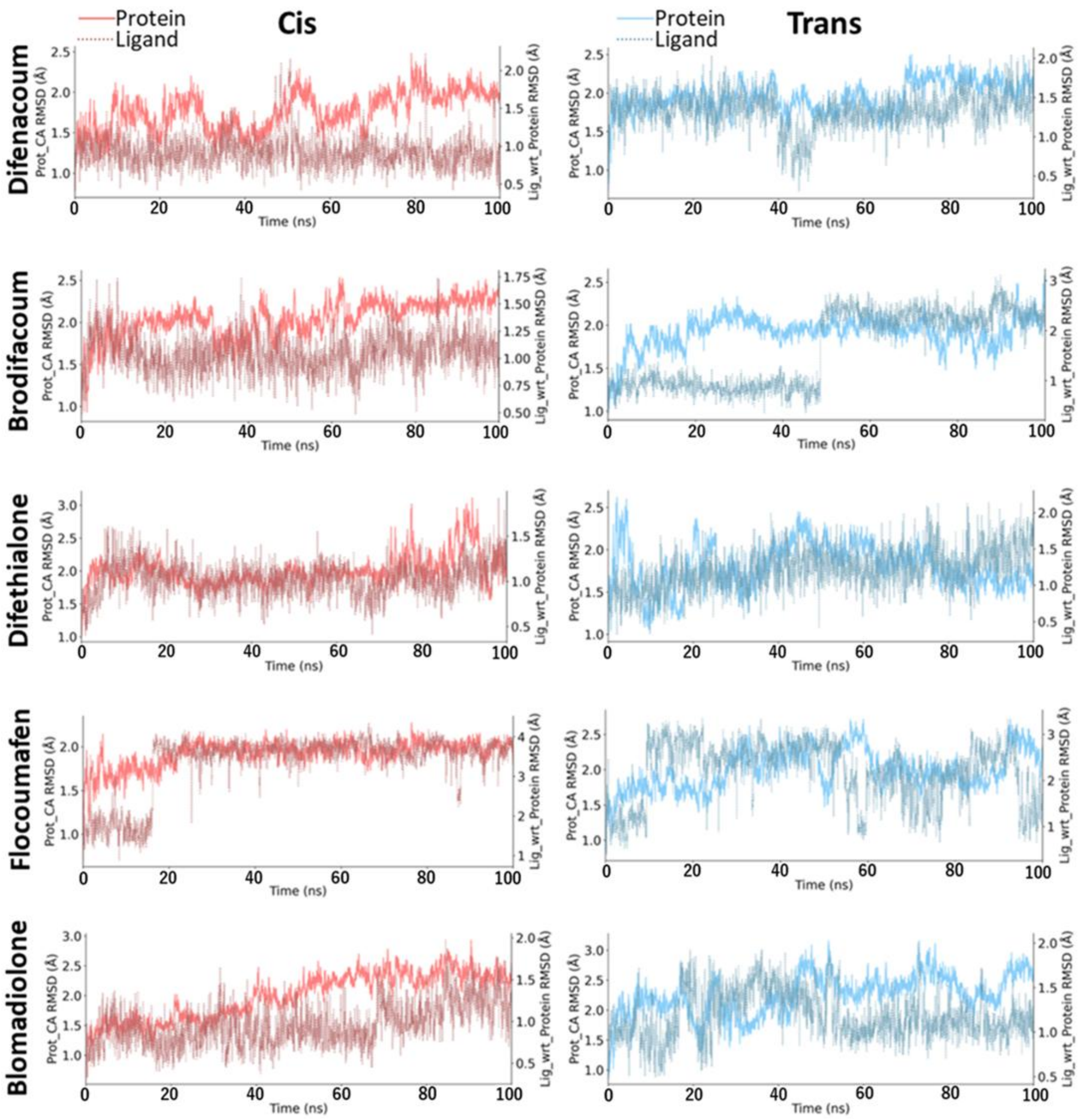

Figure S6. Protein–ligand RMSD profiles for all rat VKOR–ligand complexes. Protein–ligand RMSD plots are shown for all ten rat VKOR–ligand complexes analyzed in this study. For each compound (difenacoum, brodifacoum, difethialone, flocoumafen, and bromadiolone), the cis isomer (left panel) and trans isomer (right panel) are displayed side-by-side. The x-axis represents simulation time (0–100 ns), and the y-axis represents RMSD (nm). Each plot shows the time evolution of the protein backbone RMSD and the ligand RMSD relative to the initial minimized structure. Across all complexes, protein RMSD increased during the initial equilibration phase and subsequently reached a plateau, indicating structurally stable protein conformations during the simulations. Ligand RMSD exhibited compound- and isomer-dependent behaviors: some complexes showed low-amplitude fluctuations, whereas others displayed transient increases corresponding to ligand pose relaxation or reorientation within the binding pocket, followed by re-stabilization. Importantly, no trajectories showed unbounded RMSD divergence. Based on these observations, all simulations satisfy stability criteria defined as (i) bounded protein RMSD after equilibration and (ii) absence of continuous, unbounded ligand RMSD increase. These plots are derived directly from the complete SID reports provided in Supplementary Dataset S1.

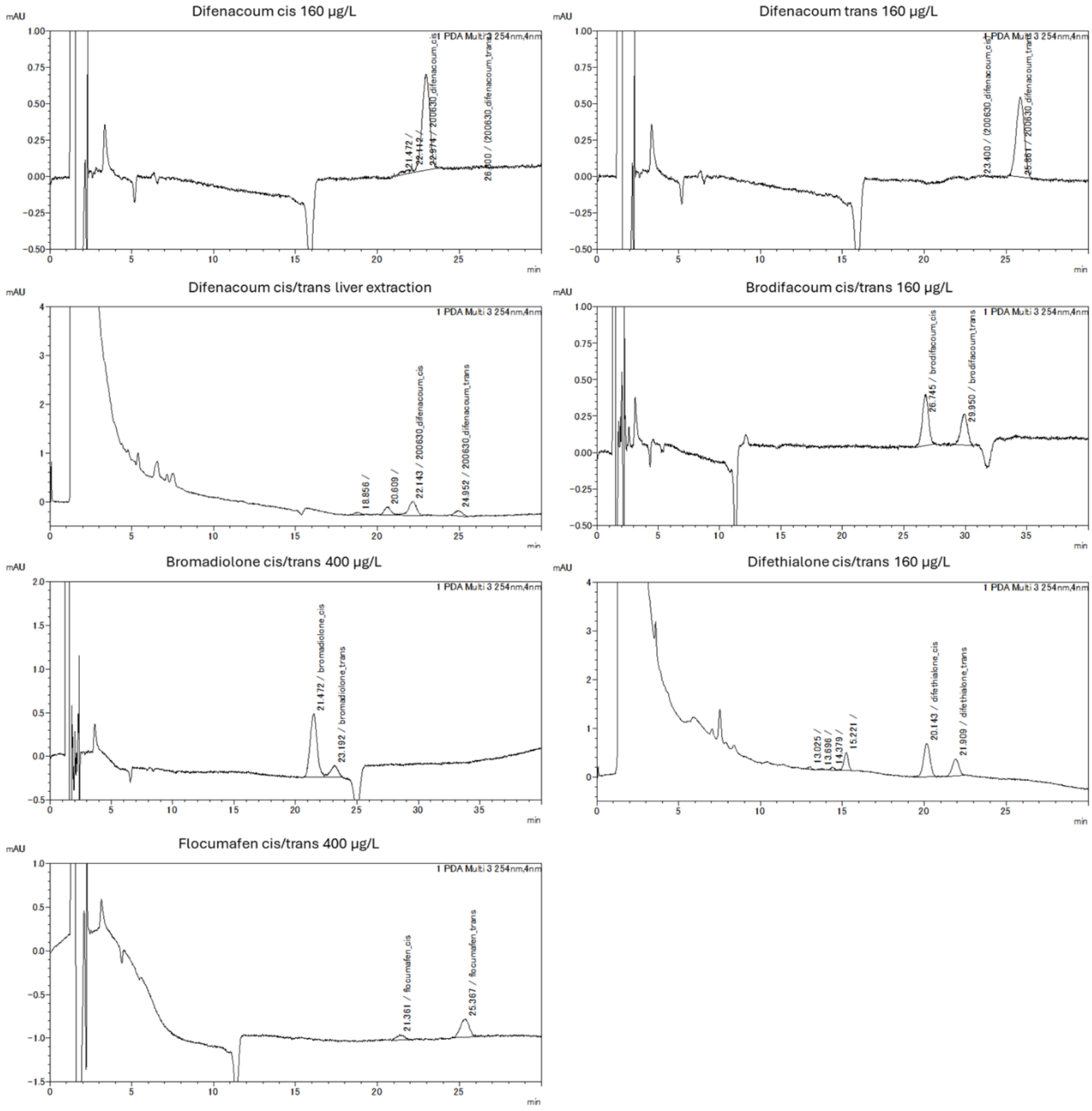

Figure S7. Representative HPLC–UV chromatograms supporting cis/trans isomer quantification of second-generation anticoagulant rodenticides (SGARs). Representative chromatograms of standard solutions are shown for difenacoum cis (160 µg/L), difenacoum trans (160 µg/L), brodifacoum cis/trans (160 µg/L), bromadiolone cis/trans (400 µg/L), difethialone cis/trans (160 µg/L), and flocoumafen cis/trans (400 µg/L), together with a representative chromatogram of a difenacoum liver extract sample. In the difenacoum liver extract chromatogram, peaks corresponding to cis- and trans-difenacoum were detected at retention times consistent with those of the corresponding standards, supporting chromatographic peak assignment in biological samples. Peak labels indicate the assigned analyte and retention time. These chromatograms illustrate chromatographic separation of the cis and trans isomers under the HPLC–UV conditions used in this study. The corresponding retention-time data, chromatographic resolution values, and calibration/linearity information are summarized in Supplementary Table S4.

Table S1. Comparison of cis/trans docking score tendencies between AF2-based VKOR models and experimentally resolved human VKOR structures

Compound	Structure	cis score	trans score	Δ(cis−trans)	Preference
	AF2 open	-13.42	-13.073	-0.347	cis
	AF2 closed	-13.007	-12.98	-0.027	cis
	PDB 6WV3	-13.055	-12.63	-0.425	cis
difenacoum	PDB 6WVH	-13.674	-13.045	-0.629	cis
	AF2 open	-13.389	-13.12	-0.269	cis
	AF2 closed	-13.011	-6.738	-6.273	cis
	PDB 6WV3	-13.031	-6.997	-6.034	cis
brodifacoum	PDB 6WVH	-13.873	-6.822	-7.051	cis
	AF2 open	-8.383	-7.001	-1.382	cis
	AF2 closed	-8.068	-6.591	-1.477	cis
	PDB 6WV3	-12.704	-13.08	0.376	trans
difethialone	PDB 6WVH	-14.282	-7.569	-6.713	cis
	AF2 open	-13.75	-13.994	0.244	trans
	AF2 closed	-13.362	-13.522	0.16	trans
	PDB 6WV3	-13.135	-13.532	0.397	trans
flocoumafen	PDB 6WVH	-14.059	-13.633	-0.426	cis
	AF2 open	-13.107	-8.797	-4.31	cis
	AF2 closed	-13.46	-8.836	-4.624	cis
	PDB 6WV3	-11.591	-10.776	-0.815	cis
bromadiolone	PDB 6WVH	-13.622	-13.142	-0.48	cis

Lower (more negative) Glide SP scores indicate more favorable predicted docking poses under the same structure-specific conditions. Δ(cis−trans) = cis score − trans score; negative values indicate cis-favored docking and positive values indicate trans-favored docking. Values are provided for qualitative comparison of cis/trans tendencies and were not interpreted as absolute binding free energies.

Table S2. Comprehensive analysis of protein–ligand interaction fraction for cis and trans isomers of second-generation anticoagulant rodenticides in complexes with rat VKOR based on PL_Contact analysis.

Difenacoum									
cis					trans				
Residue	HB	HP	PP	WB	Residue	HB	HP	PP	WB
23	0	0	0	0	23	0	0	0	0.1
26	0	33.8	0	0	26	0	15.7	0	0
30	0	0	0	1.4	30	0	0	0	0.4
54	0	37.6	0	0.1	54	0	21.2	0	21.6
55	0	28.3	48	1.4	55	0	26.4	2.7	1.4
57	0	0	0	0	57	0	0	0	0
59	0	1.3	0	0	59	0	6.1	0	0
60	0	0	0	16	60	0	0	0	2
63	0	69.6	64.4	0	63	0	62.2	65.4	0
79	0	0	0	0.3	79	0	0	0	0
80	24.3	0	0	2.6	80	46.6	0	0	0.4
81	0	0	0	29	81	0	0	0	13.1
83	0	0.4	0	0	83	0	3.1	0.2	0
86	0	0.2	0	0	86	0	0.2	0	0
87	0	3.8	29.1	0	87	0	6.9	41.1	0
112	0	22.6	0	0	112	0	28.9	0	0
117	2.2	0	0	0	117	0	0	0	0
120	0	46	0	0	120	0	39.8	0	0
123	0	1.3	0	0	123	0	3.2	0	0
124	0	0.9	0	0	124	0	6.7	0	0
128	0	4.8	0	0	128	0	14.6	0	0
134	0	20.7	0	0	134	0	8.7	0	0
135	18.4	0	0	0	135	20.4	0	0	0
139	0	0	0	0	139	100	0	0	0

Brodifacoum									
cis					trans				
Residue	HB	HP	PP	WB	Residue	HB	HP	PP	WB
22	0	0	0	0	22	0	0.2	0	0
26	0	9.6	0	0	26	0	35.6	0	0
30	0	0	0	0.1	30	0	0	0	5.6
54	0	10	0	10	54	0	28.5	0	2.2
55	0	9.7	0.8	0.2	55	0	35.8	11.5	1.5
59	0	1.4	0	1.1	59	0	3.6	0	0
60	0	0	0	1.1	60	0	0	0	9.6
63	0	62.6	31.5	0	63	0	38	77	0
79	0	0	0	0	79	0	0	0	1.1
80	70.7	0	0	3.4	80	16.3	0	0	2
81	0	0	0	7.7	81	0	0	0	23.7
83	0	34.5	0.9	0	83	0	0.6	0.9	0
86	0	0.2	0	0	86	0	0	0	0
87	0	45.7	40.4	0	87	0	7.4	78.7	0
88	0	0.2	0	0	88	0	0	0	0
112	0	26.9	0	0	112	0	2.8	0	0
117	51.2	0	0	0	117	0	0	0	0
120	0	60.7	0	0	120	0	41.4	0	0
121	0	0.3	0	0	121	0	0	0	0
123	0	0.6	0	0	123	0	5	0	0
124	0	13.2	0	0	124	0	2.1	0	0
128	0	7	0	0	128	0	17	0	0
134	0	6.5	0	0	134	0	16.7	0	0
135	6.6	0	0	0	135	24.2	0	0	0
138	48	0	0	0	138	0	0	0	0
139	0	0.2	0.1	0	139	99.9	0	0	0
142	0.1	0	0	0	142	0	0	0	0

Difethialone									
cis					trans				
Residue	HB	HP	PP	WB	Residue	HB	HP	PP	WB
22	0	0	0	0	22	0	0.1	0	0
26	0	27.9	0	0.2	26	0	23.8	0	0
30	0	0	0	24.5	30	0	0	0	10.2
54	0	26.5	0	0	54	0	17.1	0	36.3
55	0	34.7	12.4	14.8	55	0	9.6	1.4	2.2
59	0	7	0	0	59	0	6	0	0
60	0	0	0	32.3	60	0	0	0	8.2
63	0	60.4	73.1	0	63	0	20.6	83.3	0
79	0	0	0	2.3	79	0	0	0	0.6
80	3.6	0	0	5.9	80	75.6	0	0	2
81	1.1	0	0	28.9	81	0	0	0	19.8
83	0	0.3	0.1	0	83	0	0.3	0	0
86	0	0	0	0	86	0	0.1	0	0
87	0	3.5	58.2	0	87	0	10.4	72.1	0
112	0	0.4	0	0	112	0	3.7	0	0
120	0	37.9	0	0	120	0	37	0	0
123	0	8	0	0	123	0	6.2	0	0
124	0	1.8	0	0	124	0	6.7	0	0
128	0	21.8	0	0	128	0	8.8	0	0
134	0	12.9	0	0	134	0	9.4	0	0
135	41	0	0	0	135	6.2	0	0	0
138	0	0	0	0	138	2.9	0	0	0
139	100	0	0	0	139	8.6	0	0	0

Flocoumafen									
cis					trans				
Residue	HB	HP	PP	WB	Residue	HB	HP	PP	WB
22	0	0.3	0	0	22	0	0	0	0
26	0	22.3	0	0	26	0	11.2	0	0
30	0	0	0	3.7	30	0	0	0	4.4
54	0	48.6	0	0	54	0	22.8	0	46.7
55	0	29.1	23.6	0.3	55	0	20.3	1.1	1.2
59	0	1.1	0	0	59	0	6.3	0	0
60	0	0	0	4.3	60	0	0	0	2.8
63	0	31.8	17.3	0	63	0	34.8	63.4	0
79	0	0	0	1.1	79	0	0	0	0.1
80	33.5	0	0	1.2	80	40.4	0	0	0.6
81	0	0	0	42.2	81	0	0	0	28.4
83	0	1.8	1	0	83	0	6.5	1.6	0
86	0	2.3	0	0	86	0	2	0	0
87	0	23.7	0.2	0	87	0	18.7	1.4	0
90	0	0.8	0	0	90	0	0.4	0	0
112	0	45.1	0	0	112	0	32.3	0	0
113	6.2	0	0	0	113	26.4	0	0	0
120	0	55.9	0	0	120	0	44.6	0	0
123	0	6.9	0	0	123	0	7.8	0	0
124	0	1.3	0	0	124	0	4.6	0	0
128	0	5.4	0	0	128	0	11.6	0	0
134	0	4.3	0	0	134	0	16.1	0	0
135	45.6	0	0	0	135	29.3	0	0	0
139	100	0	0	0	139	100	0	0	0

Bromadiolone									
cis					trans				
Residue	HB	HP	PP	WB	Residue	HB	HP	PP	WB
26	0	38.8	0	0	26	0	29.6	0	0
30	0	0	0	0.3	30	0	0	0	4.2
54	0	35.6	0	0.2	54	0	23.2	0	3.9
55	0	20.9	32.8	0.8	55	0	16	30.6	8.9
59	0	2	0	0	59	0	1.7	0	0
60	0	0	0	8.8	60	0	0	0	36.2
63	0	97	11.1	0	63	0	82.4	53.1	0
79	0	0	0	0.1	79	0	0	0	0.9
80	45.6	0	0	2.1	80	29.7	0	0	2.2
81	0	0	0	8	81	0.2	0	0	37.6
83	0	1.8	0.7	0	83	0	4.3	1.6	0
86	0	0	0	0	86	0	0.4	0	0
87	0	1.9	19.3	0	87	0	11.8	13.4	0
112	0	8.2	0	0	112	0	11.8	0	0
116	0	0	0	0	116	0	0	0	0
117	16.7	0	0	0	117	49.3	0	0	0.2
120	0	54.7	0	0	120	0	50.5	0	0
123	0	6.3	0	0	123	0	3.5	0	0
124	0	3.5	0	0	124	0	5.6	0	0
128	0	13	0	0	128	0	16.1	0	0
134	0	5	0	0	134	0	7.8	0	0
135	55.2	0	0	0	135	23.2	0	0	0
138	92.7	0	0	0	138	20.3	0	0	0.2
139	100	0	0	0	139	96.7	0	0	0
142	57.6	0	0	0	142	2	0	0	0

Interaction frequencies (%) between ligands and protein residues were calculated based on PL_Contact analysis of 100 ns molecular dynamics simulations. Abbreviations: HB, hydrogen bond; HP, hydrophobic interaction; PP, π–π interaction; WB, water bridge.

Table S3. In silico ADMET prediction of physicochemical and metabolic parameters for cis and trans isomers of SGAR compounds using ADMET Predictor.

		CYP_iso	S+logP	S+logD	S+S_Intrins	CYPSum_CLint	CYP_HLM_CLint	CYP_RLM_CLint	CYP_MLM_CLint	N_Bonds	N_FrRotB	T_PSA
difenacoum	cis	2C9	7.623	5.433	3.81E-05	74.665	788.407	1535.382	3205.262	39	2	50.44
	trans	2C9	7.623	5.433	3.92E-05	74.665	788.407	1535.382	3205.262	39	2	50.44
brodifacoum	cis	2C9	8.134	5.792	1.57E-05	34.215	1460.509	1329.651	2290.905	40	2	50.44
	trans	2C9	8.134	5.792	1.61E-05	34.215	1460.509	1329.651	2290.905	40	2	50.44
difethialone	cis	2C9	8.16	6.65	2.02E-05	55.058	1659.007	711.575	1507.881	40	2	37.3
	trans	2C9	8.16	6.65	2.07E-05	55.058	1659.007	711.575	1507.881	40	2	37.3
flocoumafen	cis	2C9	7.932	5.669	2.69E-05	182.151	1397.113	727.868	1659.602	45	6	59.67
	trans	2C9	7.932	5.669	2.73E-05	182.151	1397.113	727.868	1659.602	45	6	59.67
bromadiolone	cis	2C9	6.775	4.669	8.78E-05	59.034	455.363	775.829	1146.164	39	5	70.67
	trans	2C9	6.775	4.669	8.72E-05	59.034	455.363	775.829	1146.164	39	5	70.67

		CYP1A2								CYP2A6				CYP2B6		
		Ind	Inh	Ki	Substr	Sites	Km	Vmax	CLint	Inh	Ki	Substr	Sites	Inh	Substr	Sites
difenacoum	cis	Yes (84%)	Yes (57%)	6.887	No (85%)	NonSub	NonSub	NonSub	NonSub	No (95%)	NonInh	No (80%)	NonSub	Yes (80%)	No (89%)	NonSub
	trans	Yes (84%)	Yes (57%)	6.887	No (85%)	NonSub	NonSub	NonSub	NonSub	No (95%)	NonInh	No (80%)	NonSub	Yes (80%)	No (89%)	NonSub
brodifacoum	cis	No (82%)	Yes (53%)	7.154	No (97%)	NonSub	NonSub	NonSub	NonSub	No (95%)	NonInh	No (80%)	NonSub	Yes (80%)	No (77%)	NonSub
	trans	No (82%)	Yes (53%)	7.154	No (97%)	NonSub	NonSub	NonSub	NonSub	No (95%)	NonInh	No (80%)	NonSub	Yes (80%)	No (77%)	NonSub
difethialone	cis	No (82%)	Yes (51%)	7.021	No (89%)	NonSub	NonSub	NonSub	NonSub	No (96%)	NonInh	No (91%)	NonSub	Yes (97%)	Yes (53%)	S2(989); C29(875); C28(805); C30(803); C19(735); C20(709)
	trans	No (82%)	Yes (51%)	7.021	No (89%)	NonSub	NonSub	NonSub	NonSub	No (96%)	NonInh	No (91%)	NonSub	Yes (97%)	Yes (53%)	S2(989); C29(875); C28(805); C30(803); C19(735); C20(709)
flocoumafen	cis	No (82%)	No (57%)	NonInh	No (97%)	NonSub	NonSub	NonSub	NonSub	No (98%)	NonInh	No (88%)	NonSub	No (65%)	No (89%)	NonSub
	trans	No (82%)	No (57%)	NonInh	No (97%)	NonSub	NonSub	NonSub	NonSub	No (98%)	NonInh	No (88%)	NonSub	No (65%)	No (89%)	NonSub
bromadiolone	cis	No (82%)	No (52%)	NonInh	No (97%)	NonSub	NonSub	NonSub	NonSub	No (95%)	NonInh	No (84%)	NonSub	Yes (87%)	No (89%)	NonSub
	trans	No (82%)	No (52%)	NonInh	No (97%)	NonSub	NonSub	NonSub	NonSub	No (95%)	NonInh	No (84%)	NonSub	Yes (87%)	No (89%)	NonSub

		CYP2C8				CYP2C9							CYP2C19						
		Inh	Ki	Substr	Sites	Inh	Ki	Substr	Sites	Km	Vmax	CLint	Inh	Ki	Substr	Sites	Km	Vmax	CLint
difenacoum	cis	Yes (99%)	0.594	Yes (93%)	C34(842); C28(830); C29(829); C27(722); C32(627); C33(627)	Yes (89%)	0.141	Yes (65%)	C29(747); C34(746); C28(731); C27(504); C32(465); C33(465)	0.948	0.87	66.987	No (52%)	NonInh	Yes (62%)	C34(718); C29(671); C28(538); C32(459); C33(459)	16.119	0.076	0.066
	trans	Yes (99%)	0.594	Yes (93%)	C34(842); C28(830); C29(829); C27(722); C32(627); C33(627)	Yes (89%)	0.141	Yes (65%)	C29(747); C34(746); C28(731); C27(504); C32(465); C33(465)	0.948	0.87	66.987	No (52%)	NonInh	Yes (62%)	C34(718); C29(671); C28(538); C32(459); C33(459)	16.119	0.076	0.066
brodifacoum	cis	Yes (99%)	0.483	Yes (93%)	C29(855); C30(847); C28(762)	Yes (89%)	0.04	Yes (65%)	C29(791); C30(789); C28(569)	0.895	0.372	30.351	No (50%)	NonInh	Yes (38%)	C30(696); C29(548)	7.856	0.036	0.065
	trans	Yes (99%)	0.483	Yes (93%)	C29(855); C30(847); C28(762)	Yes (89%)	0.04	Yes (65%)	C29(791); C30(789); C28(569)	0.895	0.372	30.351	No (50%)	NonInh	Yes (38%)	C30(696); C29(548)	7.856	0.036	0.065
difethialone	cis	Yes (99%)	0.474	Yes (93%)	C29(888); C30(835); C28(778); S2(639)	Yes (89%)	0.031	Yes (65%)	C29(913); C30(822); S2(822); C28(710)	0.946	0.7	54.013	Yes (48%)	1.196	No (68%)	NonSub	NonSub	NonSub	NonSub
	trans	Yes (99%)	0.474	Yes (93%)	C29(888); C30(835); C28(778); S2(639)	Yes (89%)	0.031	Yes (65%)	C29(913); C30(822); S2(822); C28(710)	0.946	0.7	54.013	Yes (48%)	1.196	No (68%)	NonSub	NonSub	NonSub	NonSub
flocoumafen	cis	Yes (99%)	0.297	Yes (93%)	C33(823); C31(658); C32(605); C30(516)	Yes (89%)	0.232	Yes (65%)	C33(928); C31(693); C32(671)	0.754	1.801	174.38	No (54%)	NonInh	Yes (39%)	C33(943)	5.449	0.358	0.921
	trans	Yes (99%)	0.297	Yes (93%)	C33(823); C31(658); C32(605); C30(516)	Yes (89%)	0.232	Yes (65%)	C33(928); C31(693); C32(671)	0.754	1.801	174.38	No (54%)	NonInh	Yes (39%)	C33(943)	5.449	0.358	0.921
bromadiolone	cis	Yes (99%)	0.760	Yes (93%)	C29(860); C30(853); C26(827); C27(777)	Yes (89%)	0.065	Yes (65%)	C29(848); C26(843); C30(837); C27(669)	0.99	0.707	52.1	No (52%)	NonInh	Yes (62%)	C30(759); C29(657); C26(592); C8(565); C27(445)	5.829	0.411	0.988
	trans	Yes (99%)	0.760	Yes (93%)	C29(860); C30(853); C26(827); C27(777)	Yes (89%)	0.065	Yes (65%)	C29(848); C26(843); C30(837); C27(669)	0.99	0.707	52.1	No (52%)	NonInh	Yes (62%)	C30(759); C29(657); C26(592); C8(565); C27(445)	5.829	0.411	0.988

		CYP2D6							CYP2E1			CYP3A4						
		Inh	Ki	Substr	Sites	Km	Vmax	CLint	Inh	Substr	Sites	Inh	Ki	Substr	Sites	Km	Vmax	CLint
difenacoum	cis	No (81%)	NonInh	No (69%)	NonSub	NonSub	NonSub	NonSub	No (69%)	No (98%)	NonSub	No (59%)	NonInh	Yes (72%)	C34(834); C28(715); C32(699); C33(699); C29(693); C30(507); C31(507)	72.083	4.943	7.612
	trans	No (81%)	NonInh	No (69%)	NonSub	NonSub	NonSub	NonSub	No (69%)	No (98%)	NonSub	No (59%)	NonInh	Yes (72%)	C34(834); C28(715); C32(699); C33(699); C29(693); C30(507); C31(507)	72.083	4.943	7.612
brodifacoum	cis	No (78%)	NonInh	No (71%)	NonSub	NonSub	NonSub	NonSub	No (84%)	No (90%)	NonSub	No (61%)	NonInh	Yes (73%)	C29(740); C30(703); C33(579); C34(579); C28(463)	92.475	3.165	3.8
	trans	No (78%)	NonInh	No (71%)	NonSub	NonSub	NonSub	NonSub	No (84%)	No (90%)	NonSub	No (61%)	NonInh	Yes (73%)	C29(740); C30(703); C33(579); C34(579); C28(463)	92.475	3.165	3.8
difethialone	cis	No (80%)	NonInh	No (79%)	NonSub	NonSub	NonSub	NonSub	No (97%)	No (98%)	NonSub	No (61%)	NonInh	Yes (89%)	S2(961); C29(577)	60.281	0.568	1.045
	trans	No (80%)	NonInh	No (79%)	NonSub	NonSub	NonSub	NonSub	No (97%)	No (98%)	NonSub	No (61%)	NonInh	Yes (89%)	S2(961); C29(577)	60.281	0.568	1.045
flocoumafen	cis	No (78%)	NonInh	No (68%)	NonSub	NonSub	NonSub	NonSub	No (78%)	No (83%)	NonSub	Yes (48%)	0.769	Yes (82%)	C33(987); C31(630); C32(607)	43.493	2.684	6.85
	trans	No (78%)	NonInh	No (68%)	NonSub	NonSub	NonSub	NonSub	No (78%)	No (83%)	NonSub	Yes (48%)	0.769	Yes (82%)	C33(987); C31(630); C32(607)	43.493	2.684	6.85
bromadiolone	cis	No (77%)	NonInh	No (84%)	NonSub	NonSub	NonSub	NonSub	No (85%)	No (93%)	NonSub	No (57%)	NonInh	Yes (69%)	C8(804); C26(746); C29(662); C30(644); C33(507); C34(507)	92.631	4.962	5.946
	trans	No (77%)	NonInh	No (84%)	NonSub	NonSub	NonSub	NonSub	No (85%)	No (93%)	NonSub	No (57%)	NonInh	Yes (69%)	C8(804); C26(746); C29(662); C30(644); C33(507); C34(507)	92.631	4.962	5.946

The table summarizes physicochemical properties and CYP-mediated metabolic parameters predicted using ADMET Predictor.

Abbreviations: CYP_iso: predicted cytochrome P450 isoform potentially responsible for metabolic transformation of the compound; S+logP : predicted octanol–water partition coefficient calculated using the Simulations Plus logP model; S+logD: predicted distribution coefficient at pH 7.4 derived from S+logP; S+S_Intrins: predicted intrinsic aqueous solubility (mg/mL) ; CYPSum_Clint: summed intrinsic clearance derived from CYP1A2_CLint, CYP2C9_CLint, CYP2C19_CLint, CYP2D6_CLint, and CYP3A4_CLint models; CYP_HLM_Clint: predicted molecule-level intrinsic clearance (µL/min/mg protein) for overall metabolism in human liver microsomes (HLM, unbound form); CYP_RLM_Clint: predicted molecule-level intrinsic

clearance (μL/min/mg protein) for overall metabolism in rat liver microsomes (RLM, unbound form); CYP_MLM_Clint: predicted molecule-level intrinsic clearance (μL/min/mg protein) for overall metabolism in mouse liver microsomes (MLM, unbound form) ; N_Bonds: total number of covalent bonds in the molecule; N_FrRotB: number of freely rotatable bonds excluding terminal bonds, with at least one bonded atom being sp^3-hybridized; T_PSA : topological polar surface area ($Å^2$) calculated according to the method of Ertl et al.

CYP* indicates the specific cytochrome P450 isoform indicated in the column heading. Predictions refer to human CYP isoforms unless otherwise specified. CYP*_Ind: prediction of whether the compound acts as an inducer of the indicated CYP isoform (Yes/No); CYP*_Inh: prediction of whether the compound acts as an inhibitor of the indicated CYP isoform (Yes/No); CYP*_Ki : predicted inhibition constant (Ki, μM) for the indicated CYP isoform; CYP*_Substr: prediction of whether the compound is a substrate of the indicated CYP isoform (Yes/No); CYP*_Sites: predicted atomic sites susceptible to CYP-mediated oxidation during the first metabolic step only; CYP*_Km : predicted Michaelis–Menten Km constant (μM) for CYP-mediated oxidation; CYP*_Vmax: predicted Michaelis–Menten Vmax constant (nmol/min/(nmol enzyme)) for CYP-mediated oxidation; CYP*_Clint: predicted intrinsic clearance (μL/min/mg protein) for CYP-mediated metabolism.

Table S4. Summary of HPLC-UV chromatographic conditions and analytical support for cis/trans isomer quantification

Compound	Calibration range (μg/L)	Regression equation(s) and R^2	Representative RT, min (cis / trans)	Resolution (Rs)
Difenacoum	Trans: 40.8-163.2; cis: 40.8-163.2	Trans: y = 127.880x -1224.5; R^2 = 0.9869<br>Cis: y = 135.914x -523.0; R^2 = 0.9988	24.172 / 26.160	3.7
Brodifacoum	42.4-169.6	Trans: y = 126.618x -1066.0; R^2 = 0.9972	26.745 / 29.910	3.4
Bromadiolone	416-1664	Trans: y = 84.773x -1863.0; R^2 = 0.9963	21.472 / 23.192	2.0
Difethialone	Trans: 40.8-163.2; cis: 37.6-150.4	Trans: y = 125.167x -107.0; R^2 = 0.9990<br>Cis: y = 113.269x +615.5; R^2 = 0.9991	20.143 / 21.909	2.3
Flocoumafen	408-1632	Trans: y = 22.628x +490.5; R^2 = 0.9992	21.361 / 25.367	5.7

Table S5. Comparison of computational and experimental cis/trans trends across SGARs

Compound	Hepatic residue trend (Figure 1)	VKOR inhibition (IC50) (Figure 2, Table 1)	Closed docking (Figure 4a)	Open docking (Figure 4b)	MM-GBSA (rat MD) (Figure 4c)	RMSD vs input pose (Figure S2)	Overall interpretation
Difenacoum	cis retained > trans	cis-favored, n.s.	cis-favored	cis-favored	cis-favored	cis lower (closed/open)	Broadly concordant toward a cis-favored structural/persistence tendency
Brodifacoum	No clear within-compound difference described	cis-favored	cis-favored	no clear difference	trans-favored	cis lower (closed/open)	Partially concordant; energetic rescoring disagrees with docking/IC50
Difethialone	No clear within-compound difference described	no clear difference	cis-favored	cis-favored	cis-favored	cis lower, n.s.	Broadly cis-favored in computation; experimental support limited/neutral
Flocoumafen	trans retained > cis (within-compound)	cis-favored (significant)	no clear difference	no clear difference	cis-favored	trans lower (closed/open)	Discordant between PD, PK, and different computational metrics
Bromadiolone	No clear within-compound difference described	trans-favored, n.s.	no clear difference	no clear difference	cis-favored	no clear difference	Mixed/discordant; no single metric explains behavior
